# Reaction Kinetics of the Benzylation of Adenine in DMSO: Regio‐Selectivity Guided by Entropy

**DOI:** 10.1002/cphc.202400561

**Published:** 2024-11-05

**Authors:** Dominique M. S. Buyens, Lynne A. Pilcher, Emil Roduner

**Affiliations:** ^1^ Department of Chemistry University of Pretoria Pretoria 0002 Republic of South Africa; ^2^ Institute of Physical Chemistry University of Stuttgart, D- 70569 Stuttgart Germany

**Keywords:** Adenine, Benzylation, Entropy-enthalpy compensation effect, S_N_2 reaction, Regio-selectivity

## Abstract

The factors governing the regio‐selectivity of the alkylation of adenine have been of interest for many years due to the biological importance of adenine derivatives, however, no reaction kinetic studies have been conducted. Herein, we report the rate constants and activation parameters of the benzylation of adenine under basic conditions in DMSO in the absence and presence of 15‐crown‐5 ether using real‐time ^1^H NMR spectroscopy. The reaction is second‐order for the formation of the N9‐ and N3‐benzyladenine products, with a regio‐selectivity factor 2.3 in favour of the N9‐adduct. The Gibbs free energy of activation amounts to 87±2 kJ mol^−1^ for both reactions. The formation of the N9‐adduct is more activated by 7 kJ mol^−1^, but its effect is offset by a less negative activation entropy, demonstrating that the long‐contested reason for the regioselectivity in the benzylation of adenine is dominated by compensation of entropy and enthalpy in the transition state. The kinetic parameters obtained in the presence of the 15‐crown‐5 ether indicate that the crown ether forms a complex with an adenine‐sodium ion‐pair, increasing the activation barrier. However, the Gibbs free energy in the absence and presence of the crown ether remains constant.

## Graphical abstract

The regio‐selectivity of the benzylation of adenine under basic conditions in DMSO solution in the absence and presence of 15‐crown‐5 ether is a complex interplay between all reaction system components in which a transition state entropy guides the formation of the major N9‐benzyladenine product, which otherwise experiences a 7 kJ mol^<−>1^ higher activation barrier then the minor N3‐adduct.

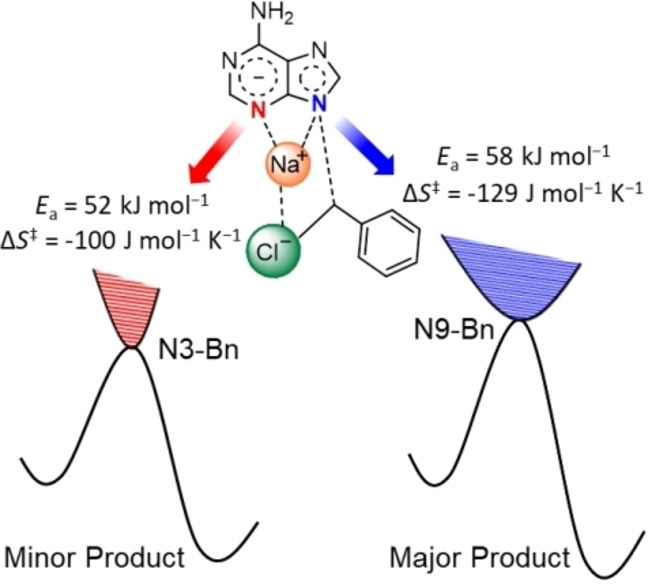

## Introduction

N9‐alkylated adenine derivatives are a useful class of therapeutics due to their biological activity; e. g. tenofovir and adefovir are N9‐alkylated adenines used in the treatment of human immunodeficiency virus[^1^, ^2^] and hepatitis B virus,[^3^, ^4^, ^5^] respectively. Other N9‐alkylated adenine derivatives have shown anti‐inflammatory activity^[6]^ and activity against diseases such as multiple sclerosis and autoimmune disease.^[7]^

The synthesis of N9‐alkylated adenine derivatives often involves the alkylation of adenine (Ade) under basic conditions using an alkyl halide in a polar aprotic solvent such as dimethylsulfoxide (DMSO) and N,N‐dimethylformamide (DMF).[^1^, ^8^, ^9^, ^10^, ^11^, ^12^, ^13^, ^14^, ^15^] Under these conditions, however, the reaction results in complicated mixtures of regio‐isomers, namely, the N9‐, N3‐, and N7‐mono‐alkylated adenine derivatives as well as possible di‐alkylated derivatives.[^10^, ^12^, ^13^, ^14^, ^16^, ^17^, ^18^, ^19^]

The causes of the regio‐selectivity in reactions of adenine have been of interest in the field of heterocyclic synthesis, and also in understanding the site preference for the alkylation of the nucleophilic atoms of nucleic acids in DNA and RNA by carcinogens and chemotherapy drugs.[^20^, ^21^, ^22^, ^23^, ^24^] The regio‐selectivity for the alkylation of heterocyclic anions has been proposed to be a result of the interplay between product stability (thermodynamic control), kinetic control, steric control, and charge control driven by electrostatics.[^16^, ^17^, ^25^, ^26^, ^27^, ^28^, ^29^, ^30^, ^31^, ^32^] A number of studies have been conducted to elucidate the factors that control the regio‐selectivity of the adeninate anion in order to increase the yield of the often desired N9‐alkylated derivative. Various factors such as product stability,^[33]^ nature of the alkylating group and the leaving group,[^16^, ^17^] steric hindrance,[^16^, ^17^] resonance stability of imidazole versus pyrimidine ring substitution,^[34]^ and the presence of the counter ion,^[17]^ have been investigated. While these factors are known to influence S_N_2 reactions[^35^, ^36^] they did not influence the resulting N‐alkylated product ratio, nor could they explain the regio‐selectivity of adenine. In contrast, the change in solvent has been reported to influence the regio‐selectivity significantly. The use or increase in the amount of polar protic solvents (water or ethanol) resulted in a substantial increase in the N3‐alkylated adenine derivative.[^17^, ^37^] The relationship between polar aprotic solvents favouring N9‐alkylated adenine and polar protic solvents favouring the N3‐alkylated adenine has been attributed to S_N_2 vs. S_N_1 solvent‐mediated control[^17^, ^37^, ^38^] or the adeninate anion accepting a proton from the polar protic solvent, yielding neutral adenine which undergoes N3‐alkylation.^[17]^ However, the product ratio within a single solvent has not been explained. A kinetic investigation would add to our fundamental understanding of the reactivity of the adeninate anion and give further insight into the regio‐selectivity at the different available nitrogen atoms.

**Figure 1:**
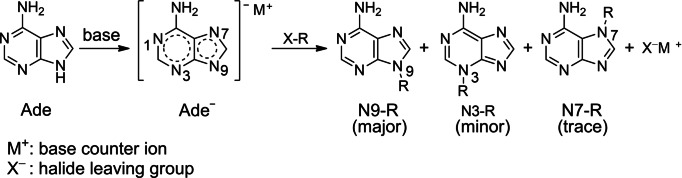
S_N_2 substitution reaction for the synthesis of N9‐alkylated adenine.

Herein we utilize real‐time ^1^H NMR spectroscopy to study the benzylation of adenine using benzyl chloride (BnCl) in the presence of sodium hydride (Figure 1). We report the kinetic parameters for the formation of the N9‐ and N3‐benzyladenine in the absence and presence of 15‐crown‐5 ether (15 C5) to investigate the effect of the Na^+^ base counter ion. In addition, we present a theoretical model of the S_N_2 mechanism for each pathway leading to N1‐, N3‐, N7‐ and N9‐benzylated adenine in the presence and absence of the sodium counter ion.

## Experimental Section

The ^1^H NMR spectra were recorded at 7.05 T (300 MHz for ^1^H) with a Bruker 300 AVANCE Ultrashield Plus. The ^1^H NMR spectra were calibrated using the DMSO‐d_6_ solvent peak at 2.50 ppm. All reagents were purchased from Sigma‐Aldrich. All samples were prepared and sealed within the glovebox using the anhydrous DMSO‐d_6_. Anhydrous DMSO‐d_6_ was prepared as follows: Powdered 4 Å molecular sieves were heated at 200 °C under vacuum for 4 days and placed inside a glovebox. The sieves were added to DMSO‐d_6_ and left for 24 hours. The solvent was analysed via ^1^H NMR to confirm the absence of water. The molecular sieves were removed by passing the solvent through a 25 mm hydrophilic polyamide syringe filter. Sodium hydride was used as a base to avoid the introduction of water into the reactions that would affect the product ratio.[^17^, ^37^, ^38^] Reagents were weighed using the Mettler Toledo XP6 Excellence Plus XP Micro Balance. The NMR tubes were placed inside the NMR spectrometer and allowed to heat up to the desired temperature of 300, 305, 310, 315 or 320 K for 10 minutes before running the kinetic experiments. All ^1^H NMR spectra were analysed and processed using MestReNova. NMR experimental setup: The ^1^H NMR spectra were collected using the zg2d pulse program in the TopSpin software (with automatic pulse calibration for the 90 degree pulse) with 0 dummy scans and the number of scans: 1, D 20: 15.5 seconds, D1: 10 seconds, an acquisition time of 5.45 seconds, and receiver gain of 90.5. The method of initial rates was used to obtain the observed rate of reaction through linear regression using GraphPad Prism 5.

For a detailed description of sample preparations for the kinetic studies, please refer to the Methods and Material section in the SI.

Concentration determination through quantitative NMR: The concentration of N9‐ and N3‐Bn and benzyl chloride during the time course of each reaction was calculated using the integration of the methyl peak of anisole and the CH_2_ peak of N9‐ and N3‐Bn and BnCl, Equation 1,

(1) $$C=CCF\times \frac{AI}{NN},$$

where C is the concentration of anisole, CCF is the concentration conversion factor, AI is the absolute integral of the methyl group integral, and NN is the number of protons.

The CH_2_ protons of N9‐ and N3‐Bn were chosen to calculate the concentration as they exhibited the shortest relaxation time out of all the product protons (Table S1 in the SI). All kinetic experiments were run at five times the longest CH_2_ T_1_ value (695 ms for N9‐benzyladenine).

### Computational Details

All electronic structure calculations were performed in Gaussian 09 rev. E.01^[39]^ (DFT). All structures were optimized at the DFT/B3LYP/6‐311++g(d,p) level of theory with empirical dispersion GD3 correction.^[40]^ The computations were performed in solvent (DMSO) using the Polarizable Continuum Model (PCM). To verify that the structures obtained at the DFT level of theory are at a minimum, frequency calculations were performed to ensure no imaginary/negative frequency was present except for the transition state structures. The pre‐reactive complexes of Ade^−^ or Na‐Ade(N3 N9) with benzyl chloride was achieved by optimizing the two structures separated by a distance of 4 Å between the CH_2_ of BnCl and the reactive nitrogen, N*X*, of the adeninate anion, either N1, N3, N7 or N9 leading to N1‐, N3‐, N7‐ or N9‐Bn, respectively. The two molecules in the adduct were scanned along the reaction coordinate (decreasing the distance between the electrophilic CH_2_ carbon on the BnCl and the reactive nitrogen by 0.1 Å steps), through a TS to the final product of each pathway. The transition state was obtained using the Berny algorithm. Frequency calculations were performed on all TS structures. Intrinsic reaction coordinate (IRC)[^41^, ^42^] calculations were performed to ensure that the transition states connected the reactants and products.

## Results

### Concentration Dependent Studies – Order of Reaction

Benzyl chloride (BnCl) was chosen to investigate the kinetics of the alkylation of adenine for several reasons. Firstly, benzyl halides can react via an S_N_2[^43^, ^44^, ^45^] or S_N_1^[46]^ mechanism. Therefore, benzyl chloride could indicate whether the regio‐selectivity between the N9‐ and N3‐alkylated adenine derivatives is controlled via the S_N_2 and S_N_1 mechanism, respectively. Secondly, BnCl reacts more slowly than benzyl bromide (BnBr) thus allowing for the study to be conducted. And lastly, the synthesis of the biologically active N9‐benzyladenine using BnCl or BnBr is often carried out when creating a library of adenine derivatives, making it an important reaction to understand. During our previous experimental studies of the alkylation of adenine using benzylic halides under basic conditions in a DMSO solution, we observed an intriguing protonation of the adeninate anion.^[47]^ The source of the proton is an alkoxy sulfonium ion intermediate, which results from the Kornblum oxidation reaction taking place between the DMSO solvent and the alkyl halide.[^48^, ^49^] To bypass the depletion of the adeninate anion starting material by protonation, the reactions were conducted in the presence of either 1,8‐Diazabicyclo(5.4.0)undec‐7‐ene (DBU) or triethylamine (TEA).

**Figure 2:**
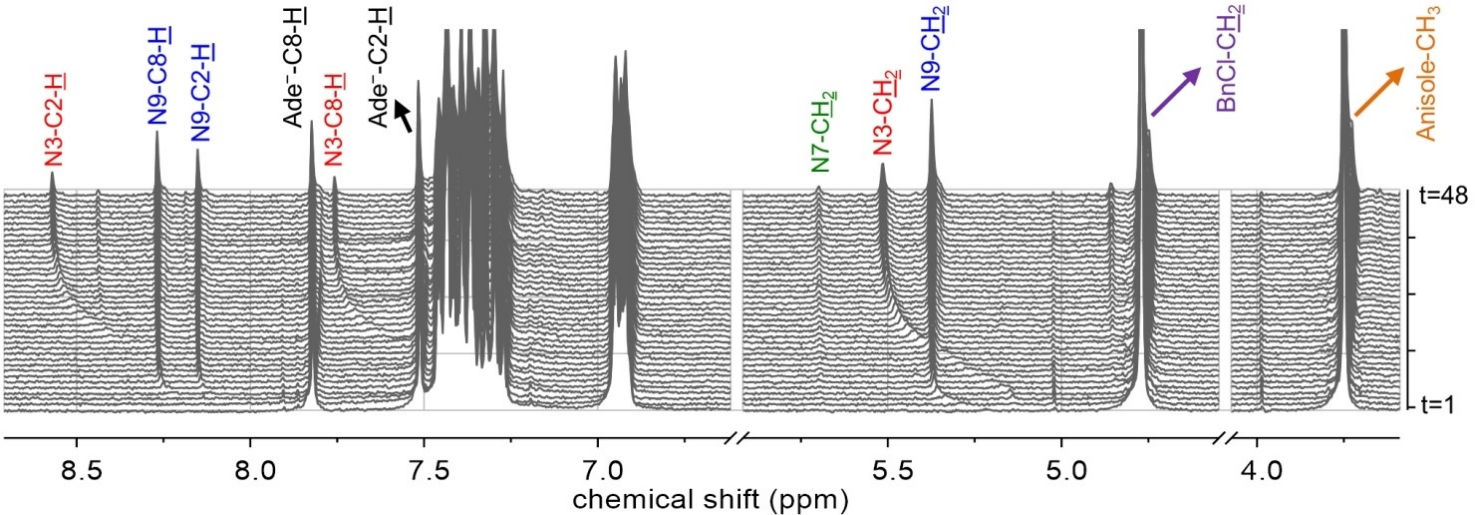
1D ^1^H NMR stacked spectra for the time course array of the reaction of the adeninate anion (13.5 mM) with BnCl (27.0 mM) in DMSO‐d_6_ and DBU (27.0 mM) at 300 K for 52 minutes (data was collected every 15.5 seconds). The proton peaks of N9‐Bn (blue), N3‐Bn (red), Ade− (black), the CH_2_ of BnCl (purple), and the CH_3_ of the internal standard anisole (orange) are labelled. Every sixth scan is shown.

Figure 2 shows a typical ^1^H NMR stacked spectra (see Figure S1 in the SI for full stacked spectra) for the time course array for the alkylation of adenine using BnCl in the presence of NaH and DBU in DMSO (See Figure S2 and S3 in the SI for TEA data). The reaction resulted in the formation of N9‐ (major), N3‐ (minor) and N7‐benzyladenine (trace amounts), in agreement with previous reports. The N9‐Bn and N3‐Bn exhibit characteristic ^1^H NMR chemical shifts of the CH_2_ group (5.36 and 5.51 ppm, respectively), and thus the appearance of the isomers could be directly monitored *in situ* by real‐time ^1^H NMR spectroscopy.

The order of the reaction at the N9 and N3 nitrogen atoms of adenine with respect to its reactants and the overall order were determined through concentration dependent studies carried out by varying the concentration of the Ade or BnCl in separate experiments. The initial concentrations of adenine ([Ade]_0_) and benzyl chloride ([BnCl]_0_) were varied from 5.58–48.5 mM and 13.8–110 mM, respectively.

**Figure 3:**
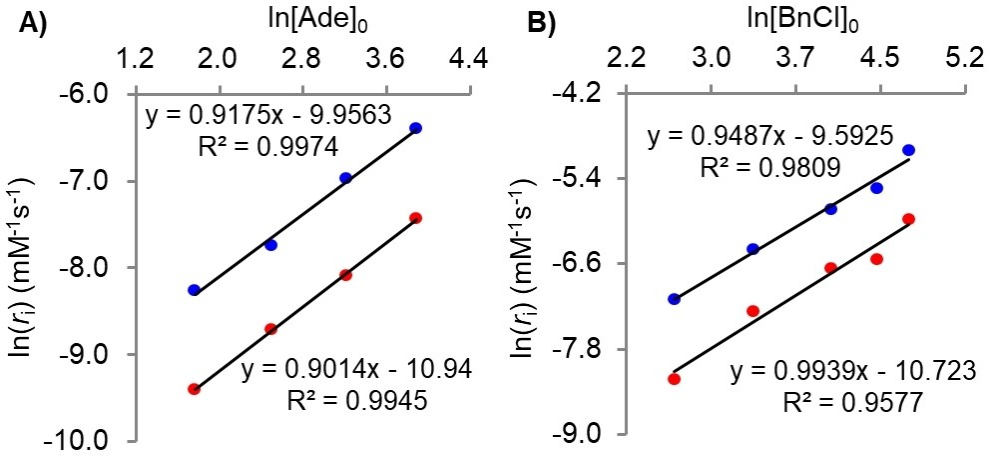
A) The ln(r_*i*_) against ln[Ade]_0_ for Ade at concentrations 5.85, 12.2, 25.0, and 48.5 mM in DMSO‐d_6_ at 295 K with BnCl and TEA at 12.9 mM, for N9‐Bn (blue circles) and N3‐Bn (red circles). B) The ln(r_*i*_) against ln[BnCl]_0_ at concentrations of BnCl:DBU (1 : 1) of 13.8, 27.6, 55.2, 82.8, and 110 mM, in DMSO‐d_6_ at 295 K with Ade at 13.5 mM, for N9‐Bn (blue circles) and N3‐Bn (red circles).

The reaction order for the alkylation of adenine at the N9 and N3 nitrogen atoms was determined using the initial rates method. The initial rate of reaction was calculated using Equation 2.

(2) $$r_{i}=\frac{\Delta [N-Bn]}{\Delta t},$$

where *r*_i_ indicates the initial rate, [N−Bn] is the experimentally determined concentration of N9‐Bn or N3‐Bn (see experimental section for details), and *t* is time in seconds. The N7‐Bn appeared in trace amounts during all concentration dependent studies at a concentration that was too low to accurately obtain rates and therefore was omitted from this study. The initial rates for the formation of N9‐ and N3‐Bn were obtained using linear regression analysis on the linear portion of the concentration versus times plot representing less than 10–20 % conversion in which the gradient was constant over the selected range. The linear fit of ln(*r*_i_) vs ln([Ade]_0_) and ln([BnCl]_0_) for N9‐ and N3‐Bn for the reaction taking place is shown in Figure 3 (refer to Figure S4 in the SI for concentration vs time plots). A slope value of near unity was obtained for the reaction at the N9 and N3 nitrogen atoms for the concentration dependence on both the Ade and BnCl. This result shows that the alkylation of adenine at N9 and N3 nitrogen atoms using BnCl follows a first‐order concentration dependence for both reagents and is second‐order overall. Therefore, the reaction at both sites follows an S_N_2 nucleophilic substitution mechanism. The same conclusions were obtained using TEA (Figure S5 in the SI) as a base, Table 1.

**Table 1:** Reaction order derived from the ln(*r*_i_) vs ln([BnCl]_0_) and ln([Ade]_0_) for N9‐Bn and N3‐Bn in anhydrous DMSO at 295 K.

Concentration varied	BnCl^[a]^		Ade^[b]^
	base present		
Regio‐isomer	DBU	TEA	TEA
N9‐Bn	0.95±0.07	0.95±0.10	0.92±0.03
N3‐Bn	0.99±0.10	1.07±0.12	0.90±0.04

[a] [Ade]=13.5 mM. [b] [BnCl]=12.9 mM.

It is interesting to note that in the ^1^H NMR stacked spectra (Figure 2), the N9‐Bn and N3‐Bn proton peaks are shielded at the very early stages of the reaction having the CH_2_ peak of both N9‐ and N3‐Bn originate from 5.13 ppm. The peaks become deshielded as the reaction continues, resulting in the final chemical shifts reported for the isolated products, i. e. the CH_2_ peak migrates to 5.36 and 5.51 ppm for the N9‐ and N3‐Bn, respectively. The migration of the proton peaks at the beginning of the reaction is not due to protonation but indicates an equilibrium process at the start of the reaction, such as the dissociation of aggregates or the dissociation of ion‐pairs. We have previously concluded that the adeninate anion does not exist in its free form in DMSO, but rather as an ion‐pair with the base counter ion, Na^+^ or K^+^, which coordinates in a bidentate fashion at the N3 and N9 nitrogen atoms of the purine ring.[^50^, ^51^] This ion‐pair formation results in a significant reorganization of the electron density within the purine ring, creating new reactive sites in comparison to its free form.^[51]^ In addition, we have shown that these ion‐pairs aggregate in DMSO.^[50]^

### Reaction Kinetics

The benzylation of adenine using BnCl in the presence of NaH in DMSO to yield N9‐Bn and N3‐Bn follows a parallel second‐order reaction, whereby the rate equation for the formation of N9‐ and N3‐Bn is given by Equation (3) and (4), respectively,

(3) $$\frac{d[N9-Bn]}{dt}=k_{9}\left[Ade\right]\left[BnCl\right],$$

(4) $$\frac{d[N3-Bn]}{dt}=k_{3}\left[Ade\right]\left[BnCl\right],$$

where *k*_9_ and *k*_3_ are the rate coefficients for the formation of N9‐ and N3‐Bn, respectively, and [Ade] and [BnCl] is the concentration of adenine and BnCl. The rate of consumption of the reagents, A (A being adenine or BnCl), is a summation of the individual rates Equation 5,

(5) $$\frac{d\left[A\right]}{dt}=-\left(k^{'}\right)\left[Ade\right]\left[BnCl\right],$$

where *k’* is the sum of *k*_9_+*k*_3_.

For first‐ and second‐order parallel reactions in which the reaction produces the kinetic products over the thermodynamic products, the product ratio (also known as the branching ratio), i. e. [N9]/[N3], is equal to the rate coefficient ratio, *k*_1_/*k*_2,_ at any time point during the reaction.[^52^, ^53^] Thus, if *k*_9_/*k*_3_ and the value of *k*_1_+*k*_2_ are known, the values of the individual rate constants can be determined.

To obtain the kinetic parameters for the formation of N9‐ and N3‐Bn, temperature dependent studies were conducted over the temperature range 300–320 K, increasing at 5 K intervals. In addition to the reaction of adenine with BnCl in DMSO in the presence of NaH with DBU as a base, a second set of experiments was carried out under identical conditions but with the addition of a 15‐crown‐5 ether (15 C5). The crown ether was added in an attempt to facilitate the removal of the base counter ion, Na^+^, which forms ion‐pairs with the adeninate anion in a DMSO solution.^[50]^ The kinetic parameters were obtained for the reaction in the presence of 15 C5 and compared with those obtained in the absence of 15 C5 to study the effect of the sodium ion on the kinetics of the reaction.

### The Rate Constants for the Formation of N9‐ and N3‐Bn in the Absence and Presence of 15‐Crown‐5 Ether

The value of the rate coefficient ratio, *k*_9_/*k*_3_, for each temperature point was calculated by plotting [N9‐Bn] against [N3‐Bn] to obtain a slope of *k*_9_/*k*_3_ (See Figure S6 in the SI). The value of *k’* was obtained from the initial rates method for the concentration dependent depletion of BnCl (refer to Figure S7 and S8 in the SI for the fitted data), monitored by the decrease in the peak intensity of the CH_2_ protons at 4.76 ppm Equation 6,

(6) $$\frac{\Delta \left[BnCl\right]}{\Delta t}=-\left(k^{'}\right){\left[Ade\right]}_{0}{\left[BnCl\right]}_{0},$$

where [Ade]_0_ and [BnCl]_0_ are the initial concentrations of adenine (6.65 mM) and BnCl (14.1 mM). The resulting rate constants, *k*_9_ and *k*_3_, are shown in Table 2. We found that similar values were obtained, within experimental error (Table S5), using the initial rates method for the formation of N9‐ and N3‐Bn.

**Table 2:** The experimentally determined rate constants (M^−1^ s^−1^) for the formation of N9‐Bn (*k_9_*) and N3‐Bn (*k_3_*), in the absence and presence of 15‐crown‐5 ether.

Temperature (K)	N9‐Bn, 10^−2^ *k*_9_	N3‐Bn, 10^−2^ *k*_3_
300	0.764±0.037	0.325±0.016
305	1.07±0.05	0.425±0.019
310	1.60±0.07	0.608±0.027
315	2.31±0.12	0.839±0.043
320	3.22±0.28	1.17±0.10
	15‐crown‐5 ether present	
300	0.637±0.039	0.274±0.017
305	1.03±0.06	0.402±0.025
310	1.67±0.09	0.601±0.034
315	2.43±0.09	0.822±0.031
320	3.72±0.35	1.21±0.11

[Ade]=6.65 mM, [BnCl]=14.1 mM.

### Arrhenius and Activation Parameters for N9‐Bn and N3‐Bn

The Arrhenius equation, Equation (7), describes the change in the rate coefficient as a function of temperature in relation to the activation energy,

(7) $$k=Ae^{-E_{a}/RT}$$

where *A* is the pre‐exponential factor and *E*_a_ is the activation energy. The Arrhenius parameters for each reaction can be obtained by plotting ln *k* against 1/T. This yields a straight line with the pre‐exponential factor, also known as the “frequency factor”, A, given by the intercept of the line at 1/T=0 and the activation energy (*E_a_*) given by the slope of the line. The kinetic data in Table 2, were used for the plot of ln *k* against 1/T (Figure S11 A and B in the SI) to determine *A* and *E*_a_ for N9‐Bn and N3‐Bn in the absence and presence of 15 C5, Table 3.

**Table 3:** Arrhenius parameters for N9‐Bn and N3‐Bn in the absence and presence of 15 C5.

	N9‐Bn		N3‐Bn	
	*E*_a_, kJ mol^−1^	log (A/M s^−1^)	*E*_a_, kJ mol^−1^	log (A/M s^−1^)
15 C5 absent	58.2±2.9	8.0±0.5	51.7±3.1	6.5±0.5
15 C5 present	70.1±3.7	10.0±0.6	58.9±6.2	7.7±1.0

In absolute rate theory, the relationship between the change in the rate coefficients, *k_1_* and *k_2_*, in Table 2 with a change in temperature over the 300–320 K range is given by the Eyring equation, Equation 8,

(8) $$k_{obs}=\frac{k_{B}T}{hc_{0}}exp\left(-\frac{{\Delta G}^{\neq }}{RT}\right),$$

which can be written in its logarithmic form,

(9) $$ln\frac{k_{obs}}{T}=\frac{{-\Delta H}^{\neq }}{RT}+ln\left(\frac{k_{B}}{hc_{0}}\right)+\frac{{\Delta S}^{\neq }}{R},$$

where *k_obs_* is the experimentally determined rate coefficient, *k*_B_ is Boltzmann's constant, *T* is the absolute temperature in degrees Kelvin, *h* is Planck's constant, *R* is the gas constant, c_0_ is 1 M when liquid standard states are used, and ▵*G* ^≠^, ▵*H* ^≠^ and ▵*S* ^≠^ are the Gibbs energy, enthalpy and entropy of activation.

The kinetic data in Table 2 were used for determining the activation parameters. A plot of the left hand side of Equation (9) vs 1/T for N9‐Bn and N3‐Bn in the absence and presence of 15 C5 (Figure S11 C and D in the SI) was used to obtain the enthalpy and entropy of activation through linear regression, Table 4.

**Table 4:** Experimental thermodynamic parameters for the formation of N9‐ and N3‐Bn in anhydrous DMSO in the absence and presence of 15 C5.

	▵*H* ^≠^, kJ mol^−1^	▵*S* ^≠^, J mol^−1^ K^−1^	${\Delta G}_{300}^{\neq }$ , kJ mol^−1^
	N9‐Bn		
15 C5 absent	56±1	−100±4	86
15 C5 present	68±2	−62±6	86
	N3‐Bn		
15 C5 absent	49±2	−129±5	88
15 C5 present	56±2	−106±6	88

The Gibbs free energy of activation, ${\Delta G}_{300}^{\neq }$ , was calculated using Equation 10,

(10) $${\Delta G}_{300}^{\neq }={\Delta H}^{\neq }-T{\Delta S}^{\neq }$$

For comparison with the Arrhenius equation (Equation (7)), ${\Delta H}^{\neq }=E_{a}-RT$ because of the extra temperature dependence in Equation (8).

### Computational Analysis of the Reaction Pathways

In order to explore and confirm the experimental thermodynamic parameters and TS structure, the pathways leading to N1‐, N3‐, N7‐ and N9‐Bn were modelled. We approached this by modelling the simplest representation of the system, involving only the adeninate anion and benzyl chloride, with DMSO included in an implicit solvent model. The system complexity was then increased by including the sodium counter ion coordinated between the N3 and N9 nitrogen atoms for all pathways (denoted as N*X*‐Bn−Na). The Na^+^ was chelated at the N3 N9 position as this was shown to be the most stable site of ion coordination in the presence and absence of explicit DMSO solvent molecules.[^50^, ^51^]

The calculated thermodynamic data and rate constants (see SI for details) for the N1‐, N3‐, N7‐ and N9‐Bn pathways in the absence and presence of the Na^+^ ion are given in Table 5.

**Table 5:** Calculated thermodynamic data and rate constants of the N1‐, N3‐, N7‐ and N9‐Bn pathways in the absence and presense of Na^+^.

	▵E_ZPVE_ (kJ mol^−1^)	▵G^≠^ (kJ mol^−1^)	Δ*S* ^≠^ J mol^−1^ K^−1^	k (M^−1^ s^−1^)
	Na absent			
N1‐Bn	33.6	81.1	−153.1	2.0×10^−3^
N3‐Bn	24.6	68.9	−141.2	2.14×10^−1^
N7‐Bn	26.7	72.5	−146.7	5.08×10^−2^
N9‐Bn	24.8	66.3	−131.4	6.08×10^−1^
	Na^+^ present			
N1‐Bn‐Na	36.6	81.5	−142.7	1.34×10^−3^
N3‐Bn‐Na	35.2	84.6	−160.4	3.78×10^−4^
N7‐Bn‐Na	29.4	76.9	−152.3	8.66×10^−3^
N9‐Bn‐Na	34.6	83.9	−160.1	5.07×10^−4^

DFT/B3LYP/6‐311++g(d,p) with empirical dispersion GD3 correction.

**Figure 4:**
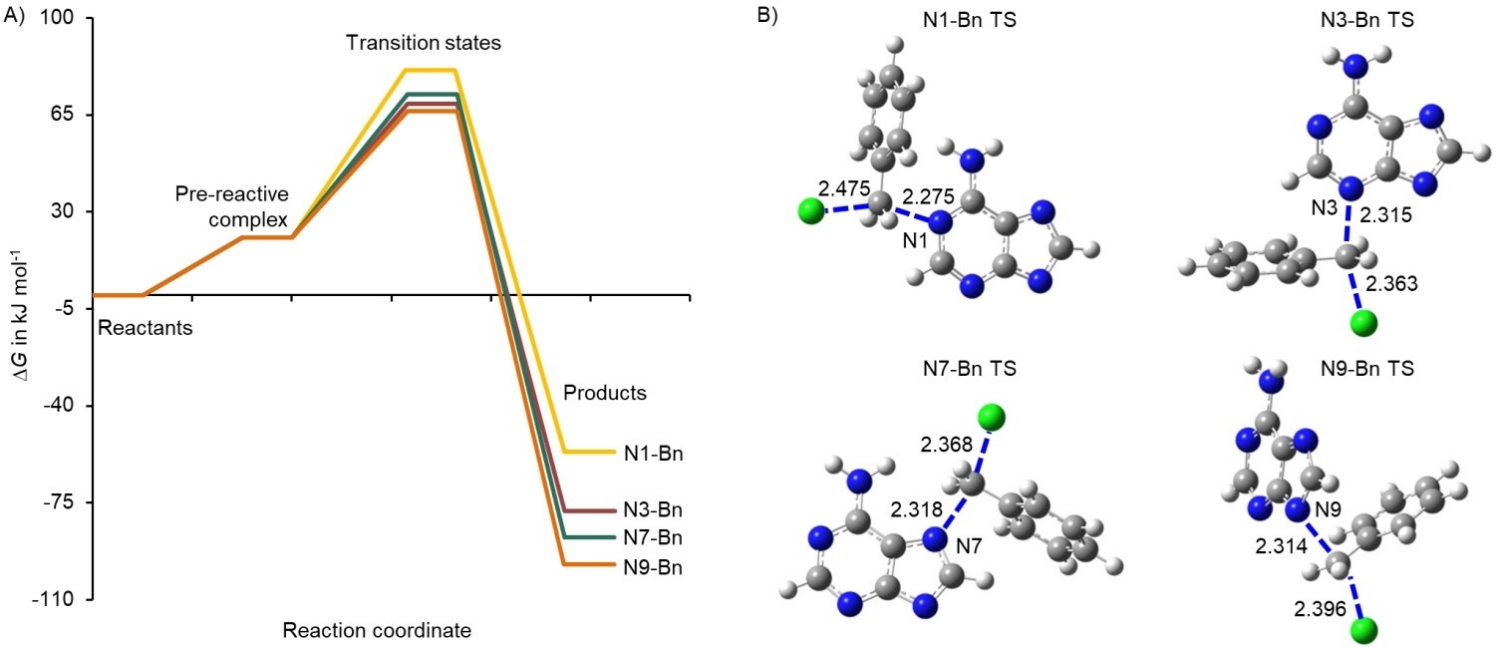
A) The ▵*G* along the reaction path for the formation of N1‐, N3‐, N7‐ and N9‐Bn, and B) the TS structures (with bond distances in Å).

**Figure 5:**
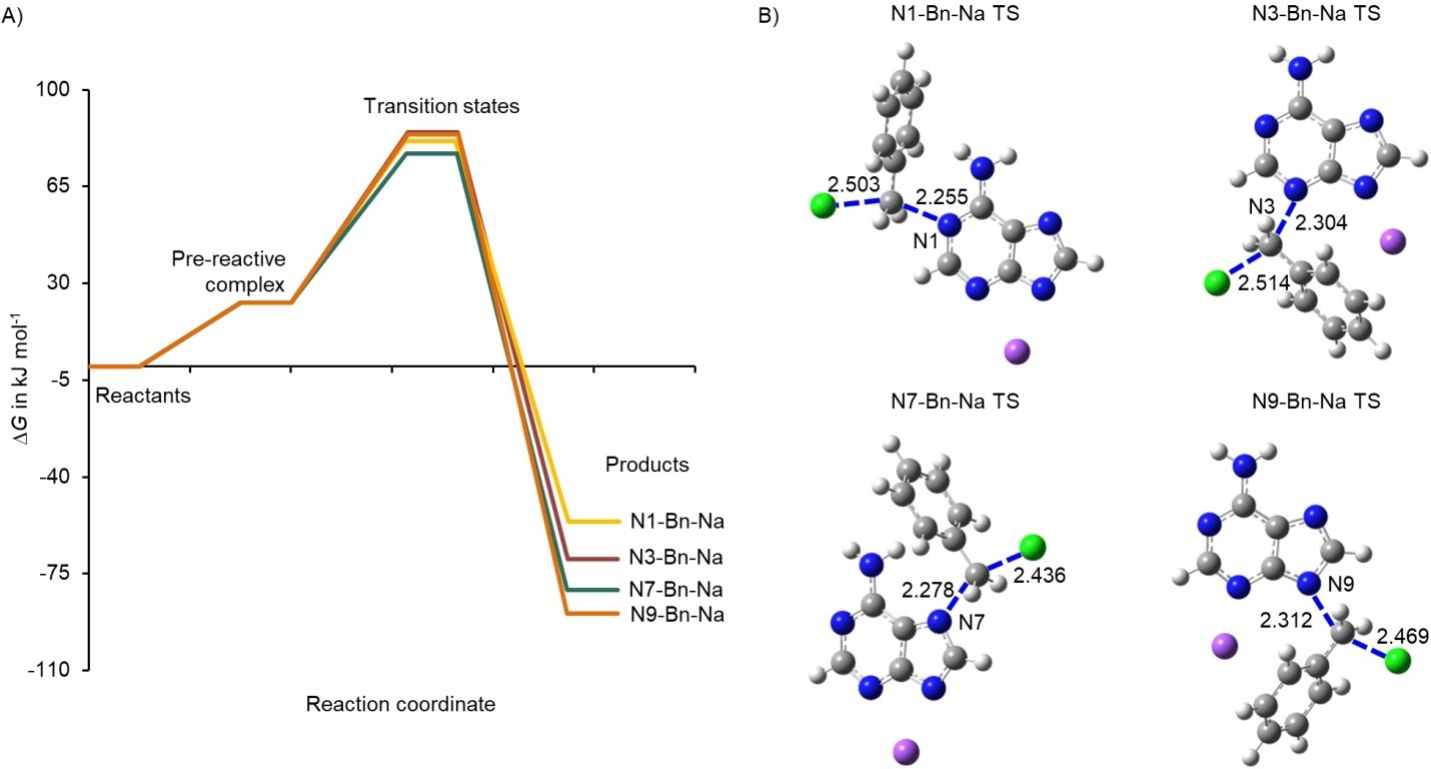
A) The ▵*G* along the reaction path for the formation of N1‐, N3‐, N7‐ and N9‐Bn−Na in the presence of the Na^+^ and B) the TS structures (with bond distances in Å).

The corresponding Gibbs free energy (▵*G*) plots and TS structures for the pathways in the absence and presence of the Na^+^ ion are shown in Figure 4 and 5, respectively (see Figure S12 for the ▵*E*_ZPVE_ plots). The reaction coordinate is defined here as the decrease in the distance between the specified N*X* atom of the purine ring and the carbon of the CH_2_ group of BnCl, d(N*X*,CH_2_). All 8 reaction pathways studied led to the formation of a typical S_N_2 TS and transfer of the benzylic group to one of the reactive nitrogen atoms of the purine ring. From Table 5, the TS electronic energies do not differ significantly for the N3‐, N9‐ and N7‐Bn pathways, i. e. less than 0.4 kcal mol^−1^. The trend in experimental ▵*G* ^≠^ values of N9‐Bn (86 kJ mol^−1^) and N3‐Bn (88 kJ mol^−1^) is well recovered by the modelling of the reaction pathways in the presence and absence of the Na^+^ ion i. e. 66.3 (N9‐Bn) and 68.9 (N3‐Bn) kJ mol^−1^ and 83.9 (N9‐Bn−Na) and 84.9 (N3‐Bn−Na) kJ mol^−1^. The product stability follows the trend of N9‐>N7‐>N3‐>N1‐Bn, both in the presence and absence of the Na^+^ ion.

## Discussion

As revealed by the rate‐concentration dependent studies, Figure 3, the benzylation of adenine under basic conditions in DMSO solutions follows an overall second‐order reaction, being first‐order with respect to adenine and benzyl chloride. The N9‐Bn is the major product, occurring at a concentration excess of 2.3 over the N3‐Bn and 12.5 over N7‐Bn. Considering the predicted product stability illustrated in Figure 4 and 5, the overall trend is N9‐>N7‐>N3‐≫N1‐Bn. The N9‐Bn is more thermodynamically favoured, being around −19.0 kJ mol^−1^ more stable than the N3‐Bn. The N7‐Bn product is more favourable than N3‐Bn by −10 kJ mol^−1^, yet it does not form in any appreciable amount in the reaction, thus suggesting that thermodynamic control is not the main driver of the regio‐selectivity. Steric hindrance is presumed to largely discourage the formation of N7‐alkylated adenine (with an exception of compounds which can form hydrogen bonds with the NH_2_ group of the adeninate anion).[^16^, ^17^, ^54^] It should be pointed out that the experimentally determined site of alkylation does not follow the order of proton tautomeric stability of neutral adenine, N9‐H>N7‐H≫N3‐H≫N1‐H[^55^, ^56^] which was predicted by computational modelling.^[33]^

### Comparison of the Activation Parameters Between N9‐Bn and N3‐Bn

The activation parameters in Tables 3 and 4 show that the resultant energy barrier (*E_a_* and ▵*H* ^≠^) for the transition state (TS) of N9‐Bn is larger than that of N3‐Bn by 7 and 12 kJ mol^−1^ in the absence and presence of the 15 C5, respectively, demonstrating that addition is not activation‐controlled. However, the activation barrier is not the only thermodynamic parameter which dictates the rate of reaction. The smaller activation energy of the N3‐Bn TS is largely counteracted by the loss of entropy which is significantly larger than that of N9‐Bn TS, with a difference in ▵▵*S* ^≠^ of almost −30 J mol^−1^ K^−1^ (▵▵*S* ^≠^ of −44 J mol^−1^ K^−1^ for the 15 C5 reaction). The ▵▵*S* ^≠^ was calculated to be −10 J mol^−1^ K^−1^ in the absence of the Na^+^ ion (Table 5) with no difference in the model with the Na^+^ ion. The latter highlights that the more complex the theoretical model becomes, the more system components need to be considered, as will be discussed further on.

**Figure 6:**
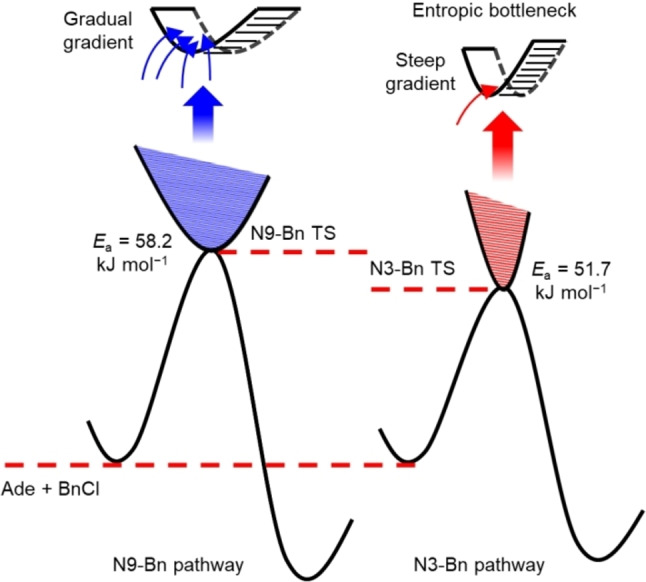
Comparison of the curvature of the PES of the N9‐Bn and N3‐Bn transition states reached. Figure adapted from Ref^[57]^_._

The larger loss of entropy for the N3‐Bn TS shows that the system is tighter and becomes more highly ordered compared to the N9‐Bn TS. This suggests that the progression from the N3‐Bn TS to the product is restricted by entropy. Figure 6 is an illustration of the potential energy surface (PES) of N9‐ and N3‐Bn in which an entropic bottleneck exists at the TS region of N3‐Bn. In terms of energy states, the shape of the PES at the TS region is indicative of the population of different energy levels resulting from the number of paths leading to the TS. A steeper curvature of the TS region indicates fewer accessible states, a concept discussed by E. G. Lewars.. ^[57]^ Hence, if we consider the N9‐Bn and N3‐Bn TS regions, although the N9‐Bn TS has a higher *E_a_* than the N3‐Bn TS, more molecules have sufficient energy to pass through the N9‐Bn TS region at any given temperature as compared to N3‐Bn TS. This results in a more favourable ${\Delta G}_{300}^{\neq }$ value (86 kJ mol^−1^) for the formation of N9‐Bn, being 2 kJ mol^−1^ lower in energy than for N3‐Bn, 88 kJ mol^−1^. A difference in ▵*G* ^≠^ of 2 kJ mol^−1^ leads to an increase in the rate constant ratio of 2.5, which is close to ratio of 2.3–2.5 obtained experimentally for the rates of formation of N9‐Bn to N3‐Bn at 300–305 K (Figure S6). The calculated rate constant of the N9‐Bn pathway is 2.8 times larger than that of N3‐Bn and 12 times larger than that of N7‐Bn (12.5 obtained experimentally). The lack of N1‐Bn product formation from experimental work is supported by the extremely large calculated N9/N1 ratio of 391.

In the modelled system, the N9‐Bn pathway is the most exothermic reaction, which in accordance with Hammonds postulate, will have an earlier, and thus looser, TS.^[58]^ Based on the difference in the activation entropies, and from Equation (10), lower temperatures will lead to an increase in the amount of N3‐Bn, and potentially invert the selectivity of the reaction. Figure S6 in the SI shows a decrease in the ratio of [N9‐Bn]/[N3‐Bn] as the temperature decreases from 320–300 K.

From the theoretical investigation the N1‐Bn TS has the largest energy barrier (▵*G* ^≠^ is 15 kJ mol^−1^ larger than N9‐TS) in the absence of the Na^+^ ion. The lowest energy barrier is for the N9‐Bn TS, being 3 and 6 kJ mol^−1^ lower in energy than the N3 and N7 TS, respectively. However, for the Na^+^ complexed TSs, the N7‐Bn−Na pathway has the lowest energy barrier, being around −7.0 kcal mol^−1^ lower than N9‐ and N3‐Bn. This is a result of the Na^+^ ion in the immediate reactive site and hence, represents the barrier to the dissociation of the ion‐pair by the incoming electrophile without the assistance of the solvent. In addition, the repulsion between the positive charge on the Na^+^ ion and the developing positive charge of the incoming electrophile will contribute to the increase in activation energy. The presence of ion pairs, having the ion association largest for Li^+^>Na^+^>K^+^, has been shown to decrease the reaction rates with a decrease in cationic radius for enolate anions,[^59^, ^60^]

The effect of including explicit solvent molecules to assist with ion‐pair dissociation was studied by performing a scan in which the Na^+^ ion chelated at the N3 N9 position of Ade^−^ is moved away from the adeninate anion. This was done with DMSO included as an implicit and an explicit (incorporating 4 explicit DMSO solvent molecules) solvent system (details are discussed in the SI, see Figure S13 and S14). The molecular system experiences an energy penalty of 43 kJ mol^−1^ for ion‐pair dissociation using the implicit model. The inclusion of explicit DMSO solvent molecules reduces the energy required for the dissociation of the Na^+^ ion by −28 kJ mol^−1^ relative to the system studied using implicit DMSO solvent. This is in line with previous research which showed that the inter‐ionic interactions between Na^+^ and Ade^−^ are weakened in the presence of explicit DMSO solvent molecules.^[51]^ Hence, if the benzylation of the adeninate anion is to be studied in the presence of the Na^+^ counter ion, the DMSO molecules will need to be included as they will greatly reduce the amount of energy required to shift the Na^+^ ion out of place as the electrophile is approached by N3 and N9 atoms of the purine ring.

### Comparison of the Entropy of Activation of N9‐Bn and N3‐Bn With that of s_n_2 Reactions

While it is common to analyse kinetic activation energies, the pre‐exponential factors are much less often used although Eyring's absolute rate theory provides a strong tool for their interpretation.^[46]^ From Equation (9), the intrinsic pre‐exponential factor, ${log(k}_{B}T/hc_{0})$ =12.8, and a deviation from this value by one unit corresponds to a change in activation entropy, ▵*S* ^≠^, by 19.1 J mol^−1^ K^−1^. The experimental values in Table 3 span a range between 6.5 and 10.0, revealing activation entropies between −120 J mol^−1^ K^−1^ and −53 J mol^−1^ K^−1^.

The ▵*S* ^≠^ for bimolecular nucleophilic S_N_2 reactions is always negative as the TS is well organised and results in a loss of three translational (estimated around −159 J mol^−1^ K^−1^) and two to three rotational degrees of freedom.^[61]^ However, the experimental value for the loss of entropy for both the N9‐ and N3‐Bn is smaller than that expected for an S_N_2 mechanism, i. e. ▵*S* ^≠^ being −100 and −129 J mol^−1^ K^−1^ (Table 4) respectively. Although this could mean a “looser” TS, in particular that of the N9‐adduct, the computational analysis of the TS structures, Figure 4 and 5, indicate “tight” TSs, i. e. the bond distance between the bonding centres of the N*X*, CH_2_ and Cl in the TS are extended by around 1.5–1.7 times the equilibrium bond distance in the product and reagent and thus can be described as “tight”. A “loose” TS has the bond distance extended by 2.9±0.2 times the length of the stable bond distance in the reactant and product i. e. for N, O and C atoms this extend in bond length is around 4–4.5 Å.^[61]^ Hence, other factors which contribute to the smaller loss of entropy of activation need to be considered, such as the depletion of bonding electrons between the atoms involved in the transfer of the molecular group, as well as antibonding interactions between electron pairs.^[62]^ Additionally, the change in charge distribution of the molecules in the TS conformation can lead to changes in the solvent shell from i) reorganization of the solvent molecules around the TS and ii) changes in the strength of interaction between the TS molecules with the solvent and therefore contribute to an increase or decrease in the entropy of activation.[^62^, ^63^, ^64^] The less than maximum value of ▵*S* ^≠^ of both the N9‐ and N3‐Bn can result from a combination of the above factors. Furthermore, previous research showed that the adeninate anion in DMSO does not exist as its free anion, but rather as an ion‐pair with the base counter ion, Na^+^ in this study, which is coordinated in a uni‐ or bi‐dentate fashion at the N9 and N3 nitrogen atoms of the adenine purine ring.[^50^, ^51^] Theoretical models suggested that the DMSO solvent molecules interact with the NH_2_ group of the adeninate anion via hydrogen bonding. This interaction has a significant amount of electron density shared between the DMSO molecules and the adeninate anion molecule, resulting in a large covalent component of the interaction.^[51]^ Therefore, the smaller loss of entropy for the benzylation of the adeninate anion at N9/3 (Table 4) could be attributed to the liberation of the Na^+^ base counter ion and the weakening of the interaction between the DMSO solvent molecules and adenine due to the change in the charge on the adeninate anion at the TS.

### The Effect of 15c5 on the Thermodynamic Parameters

The presence of the 15 C5 increases the *E_a_* and ▵*H* ^≠^ of the reaction (Tables 3 and 4) by 12 and 7.0 kJ mol^−1^ for the N9‐ and N3‐Bn TS, respectively. The 15 C5 has been proposed to complex to the Na‐Ade complex without fully removing the counter ion.^[50]^ A simultaneous complexation of the Na^+^ to the Ade^−^ and 15 C5 would result in steric hindrance for the incoming benzyl chloride and consequently an increase in *E_a_*. The observed increase (less negative) in the ▵*S* ^≠^ term of 38 and 23 J mol^−1^ K^−1^ for the N9‐ and N3‐Bn TS, can be attributed to the liberation of the 15 C5‐Na^+^ complex and the DMSO solvent molecules. Although there is an increase in the enthalpy and entropy of activation for both the N9‐ and N3‐Bn TS in the presence of 15 C5, the overall ${\Delta G}_{300}^{\neq }$ remains constant, being 86 and 88 kJ mol^−1^, respectively. This could indicate a possible entropy‐enthalpy compensation effect,^[65]^ however, this will need to be confirmed with further experimental work (refer to SI for a more detailed discussion, Figures S15–S17).

## Conclusions

Regio‐selectivity in the alkylation of adenine has been poorly understood despite several investigations into the causes during the synthesis of bioactive compounds or in the actions of carcinogens or chemotherapy drugs. By studying the kinetics of the benzylation of the adeninate anion by varying the concentrations of the reactants and the temperature of the reaction we have provided new insights into the mechanisms for the formation of the two dominant products, the N9‐ and N3‐benzyladenine and the origins of the regio‐selectivity.

We have shown that the benzylation of adenine in DMSO is kinetically of first order with respect to the adeninate and the benzyl chloride, corresponding formally to second‐order (S_N_2 mechanism) for the formation of both regio‐isomers, N9‐Bn and N3‐Bn. However, the crown ether and the DMSO solvent are also involved in the reaction complex, but their additional contribution to the reaction order was not investigated. Through temperature dependent studies, the activation parameters for the formation of N9‐ and N3‐Bn revealed a higher activation barrier for N9‐Bn, which is the major product of the reaction. However, this increase in the barrier height is largely compensated for by the increase in the entropy at the transition state. The regioselectivity of a factor 2.3 corresponds to a Gibbs free energy of activation for the formation of N9‐Bn that is lower by 2 kJ mol^−1^ than for N3‐Bn. Based on the differences in the activation entropies, it is predicted that the selectivity will invert at lower temperatures and that the presence of higher concentrations of the 15 C5 will enhance this effect.

The kinetic parameters obtained in the presence of the 15 C5 support previous studies which suggest that the 15 C5 forms a stabilizing complex with the adeninate anion and the Na^+^ counter ion‐pair instead of fully removing Na^+^, as evidenced by the increasing activation barrier. However, the overall Gibbs free energy remains approximately constant, both in the absence and presence of the 15 C5, providing evidence for a second, smaller entropy‐enthalpy compensation effect. This may indicate that the complexed sodium ion is less potent to abstract the chloride ion from benzyl chloride. This highlights the property of the system that is dependent on the intermolecular interactions between the reacting components, the counter ions and the solvent. Unless the properties of the counter ions or the solvent change significantly, there is little leverage for external control of regio‐selectivity.

This study was conducted using a single alkylation agent (BnCl) and a single solvent (DMSO). Future research into the regio‐selectivity of the alkylation of adeninate could explore the effect of various solvents, which have been shown to influence the regio‐selectivity, as well as different alkylating groups on the kinetic parameters to investigate the enthalpy‐entropy effect in related reaction systems.

## Conflict of Interests

The authors declare no conflict of interest.

1

## Supporting information

As a service to our authors and readers, this journal provides supporting information supplied by the authors. Such materials are peer reviewed and may be re‐organized for online delivery, but are not copy‐edited or typeset. Technical support issues arising from supporting information (other than missing files) should be addressed to the authors.

Supporting Information

## Data Availability

The data that support the findings of this study are available from the corresponding author upon reasonable request.
