# Supplementary material for: Reaction Kinetics of the Benzylation of Adenine in DMSO: Regio‐Selectivity Guided by Entropy
Source: Chemphyschem. 2024 Nov 5;25(23):e202400561. doi: 10.1002/cphc.202400561 (PMC11614369; doi:10.1002/cphc.202400561)
Supplement: Supplementary file 1 — Supporting Information [file CPHC-25-e202400561-s001.pdf]

ChemPhysChem

Supporting Information

Reaction Kinetics of the Benzylation of Adenine in DMSO: Regio-Selectivity Guided by Entropy

Dominique M. S. Buyens,* Lynne A. Pilcher, and Emil Roduner

REACTION KINETICS OF THE BENZYLATION OF ADENINE IN DMSO: REGIO-SELECTIVITY GUIDED BY ENTROPY

Dominique M.S. Buyens[1]*, Lynne A. Pilcher[1], and Emil Roduner[1,2]

[1] Department of Chemistry, University of Pretoria, Pretoria 0002, Republic of South Africa, [2] Institute of Physical Chemistry, University of Stuttgart, D-70569 Stuttgart, Germany

Supporting Information

Table of contents

Contents

Materials and Methods 1

1D ^{1}H NMR stacked spectra for the benzylation of adenine in the presence of DBU...... 4

1D ^{1}H NMR stacked spectra for the benzylation of adenine in the presence of TEA 5

Concentration dependent studies – Order of reaction... 6

Reaction kinetics – Temperature dependent experiments 10

Arrhenius and activation parameters for N9-Bn and N3-Bn 16

Computational analysis of the reaction pathway 17

The entropy-enthalpy compensation effect 21

References... 25

Materials and Methods

Adenine concentration dependent study: A stock solution was prepared within the glovebox: NaH (7.35 mg, 0.306 mmol) was added to a heterogeneous mixture of adenine (34.5 mg, 0.255 mmol) and anhydrous DMSO-d_6 (3945 μl). The solution was stirred until bubbles of H_2 gas ceased. Anisole (55 μl, 0.506 mmol) was added as an internal standard. Solutions for the adenine concentration dependent study were prepared with a final adenine concentration of A (5.85), B (12.2), C (25.0) and D (48.5 mM). The concentration was adjusted by the addition of dried DMSO-d_6 to a final volume of 400 μl. To each NMR tube, triethylamine (TEA) was added (1.5 μl, 0.011 mM). The NMR tubes were sealed and removed from the glovebox. The reactions were initiated by the addition of BnCl (0.6 μl, 0.005 mmol, 12.9 mM). The NMR tubes were sealed, shaken, and inserted into the spectrometer. The ^{1}H NMR spectra were immediately recorded at 295 K, taking about a minute to obtain the first spectrum for each sample. Scans were collected every 15.5 seconds for 52 minutes.

Benzyl chloride concentration dependent studies: A stock solution was prepared within the glovebox: NaH (6.39 mg, 0.266 mmol) was added to a heterogeneous mixture of adenine (30.0 mg, 0.222 mmol) and anhydrous DMSO-d_6 (5000 μl). Anisole (24.7 μl, 0.227 mmol) was added as an internal standard. Solutions for the benzyl chloride concentration dependent studies were prepared from the stock solution with an adenine concentration of 13.5 mM in NMR tubes in a final volume of 400 μl using DMSO-d_6 for dilution. Each reaction contained 13.6 mM of anisole as an internal standard. To each

tube, either A) 1,8-Diazabicyclo(5.4.0)undec-7-ene (DBU) at 1 (0.81 μl, 0.005 mmol), 2 (1.61 μl, 0.011 mmol), 4 (3.22 μl, 0.022 mmol), 6 (4.84 μl, 0.032 mmol), or 8 equivalents (6.44 μl, 0.043 mmol), or B) TEA at 1 (0.75 μl, 0.005 mmol), 2 (1.50 μl, 0.011 mmol), 4 ((3.00 μl, 0.022 mmol)), 6 (4.50 μl, 0.032 mmol), or 8 equiv (6.00 μl, 0.043 mmol), was added. The NMR tubes were sealed and removed from the glovebox. The reactions were initiated by the addition of 1 (0.62 μl, 0.005 mmol), 2 (1.24 μl, 0.011 mmol), 4 (2.48 μl, 0.022 mmol), 6 (3.73 μl, 0.032 mmol), or 8 equiv. (4.97 μl, 0.043 mmol) of benzyl chloride to the corresponding equiv. of DBU or TEA for a concentration profile (A) 13.8, (B) 27.6, (C) 55.2, (D) 82.8 and (E) 110 mM of BnCl. The ^{1}H NMR spectra were recorded immediately at 295 K, taking about a minute to obtain the first spectra. Scans were collected every 15.5 seconds for 52 minutes.

Temperature dependent chemical kinetics for the benzylation of the adenine: A stock solution was prepared by adding NaH (3.62 mg, 0.151 mmol) to a heterogeneous mixture of adenine (18.5 mg, 0.137 mmol) in 2500 μl of dried DMSO-d_6. The stock was split into 2 volumes (1250 μl each) in which one of the two volumes had 15-crown-5 ether added (18.0 μl, 0.091 mmol) and the other volume had 18.0 μl DMSO-d_6 added. Anisole (7.5 μl, 0.069 mmol, 1.0 eq.) and DBU (11 μl, 0.074 mmol, 1.1 eq.) were added to each 1250 μl stock. For each sample, 50 μl of the stock volume was added to 350 μl of DMSO-d_6 to have a final volume of 400 μl. For each NMR kinetic run, BnCl (0.65 μl, 14.01 mM, 2.1 eq.) was added to the NMR tube, mixed and the ^{1}H NMR data was immediately recorded. Three replicates were run for each temperature selected: 300, 305, 310, 315 and 320 K. Each reaction had a final concentration of adenine (6.65 mM),

anisole (6.69 mM), DBU (7.14 mM) and BnCl (14.1 mM) for each ^{1}H NMR kinetic run. The reactions with the 15-crown-ether had a final concentration of 15C5 of 8.82 mM.

The T_1 relaxation times of Na-Ade, N9- and N3-benzyladenine in DMSO-d_6 at 295 K are given in Table S1.

Table S1: Proton T_1 relaxation times of Na-Ade, N9- and N3-benzyladenine in DMSO-d_6 at 295 K.

	NH_2	C2-H	C8-H	CH_2
Na-Ade	642 ms	7.989 s	4.657s	-
N9-benzyladenine	-	8.731 s	4.089s	695 ms
N3-benzyladenine	1.121 s	1.897 s	698 ms	509 ms

1D ^{1}H NMR stacked spectra for the benzylation of adenine in the presence of DBU

Full 1D ^{1}H NMR stacked spectra for the benzylation of adenine using BnCl in the presence of DBU in dried DMSO are shown in Figure S1.

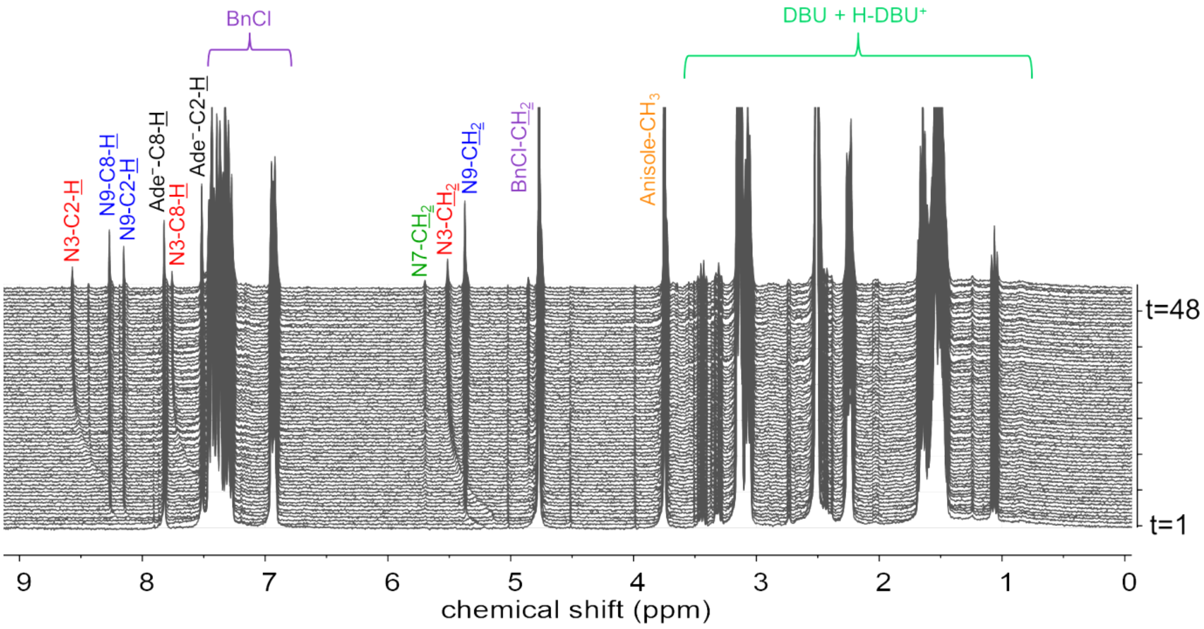

Figure S1: Full 1D ^{1}H NMR stacked spectra for the time course array of the reaction of the adeninate anion (13.5 mM) with BnCl (27.0 mM) in DMSO-d_6 and DBU (27.0 mM) at 300 K for 52 minutes (data was collected every 15.5 seconds). The proton peaks of N9-Bn (blue), N3-Bn (red), Ade$^-$ (black), the CH_2 of BnCl (purple), the CH_3 of the internal standard anisole (orange), and DBU and protonated DBU, H-DBU$^+$, (green) are labelled. Every fourth scan is shown.

1D ^{1}H NMR stacked spectra for the benzylation of adenine in the presence of TEA

Full 1D ^{1}H NMR stacked spectra for the benzylation of adenine using BnCl in the presence of TEA in dried DMSO are shown in Figure S2, and the zoomed in version from the 4-9 ppm range is shown in Figure S3.

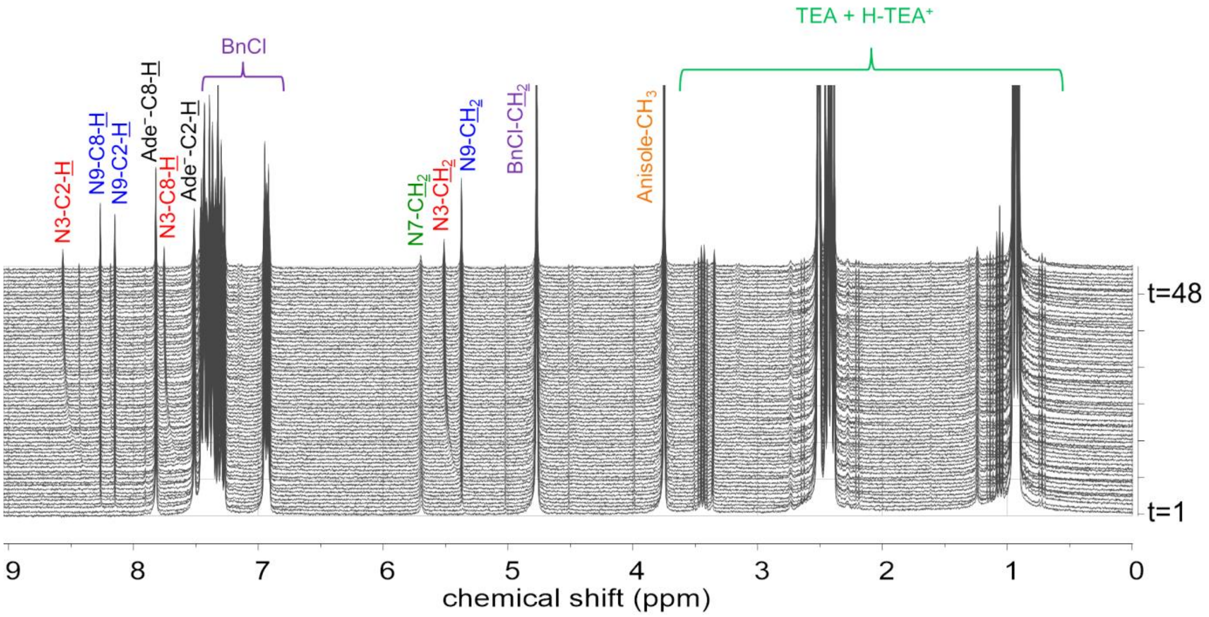

Figure S2: Full 1D ^{1}H NMR stacked spectra for the time course array of the reaction of the adeninate anion (13.5 mM) with BnCl (27.0 mM) in DMSO-d_6 and TEA (27.0 mM) at 300 K for 52 minutes (data was collected every 15.5 seconds). The proton peaks of N9-Bn (blue), N3-Bn (red), Ade$^-$ (black), the CH_2 of BnCl (purple), the CH_3 of the internal standard anisole (orange), and TEA and protonated TEA, H-TEA$^+$, (green) are labelled. Every fourth scan is shown.

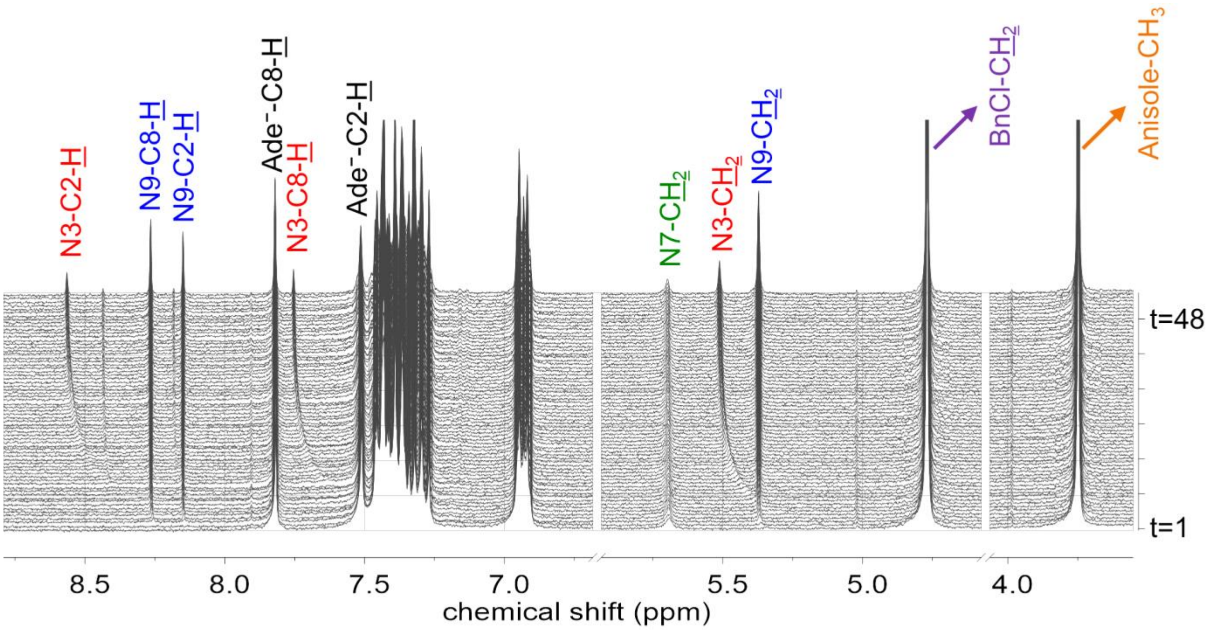

Figure S3: Zoomed in 1D ^{1}H NMR stacked spectra for the time course array of the reaction of the adeninate anion (13.5 mM) with BnCl (27.0 mM) in DMSO-d_6 and TEA (27.0 mM) at 300 K for 52 minutes (data was collected every 15.5 seconds). The proton peaks of N9-Bn (blue), N3-Bn (red), Ade$^-$ (black), the CH_2 of BnCl (purple), and the CH_3 of the internal standard anisole (orange), are labelled. Every fourth scan is shown.

Concentration dependent studies – Order of reaction

The concentration vs time plots for the rate dependent formation of N9-Bn and N3-Bn on the concentration of adenine (Figure S4 A and B) and BnCl (Figure S4 C to F) is shown below. The gaps in the plots represent the area in which the peaks of the CH_2 of N9- and N3-Bn crossed each other during the experiment. These points were deleted and data was processed to obtain rates via the initial rates method, Table S2 – S4. Figure S5 shows the linear fit of ln(r_i) vs ln($[BnCl]_0$) in the presence of TEA for N9- and N3-Bn. The formation of N3-Bn in the presence of TEA, Figure S4 D, shows an induction period. This could be artificially introduced due to the detection limit of the

spectrometer, or, could indicate a slightly slower reaction due to the competing Kornblum oxidation reaction, in which TEA does not fully prevent the conversion of deprotonated adenine to adenine, thus leading to less starting material.

Table S2: The rates (mM s^{-1}) of reaction for N9- and N3-Bn obtained with increasing concentration of adenine, using the initial rates method. The mole ratio of TEA:BnCl is 1:1, with [BnCl] = 12.9 mM, in DMSO-d_6 at 295 K

	N9-Bn		N3-Bn	
[Ade] (mM)	$\times 10^{-3}$ mM s^{-1}	R^2	$\times 10^{-4}$ mM s^{-1}	R^2
5.83	0.259 ± 0.004	0.9824	0.828 ± 0.027	0.93935
12.2	0.435 ± 0.008	0.96725	1.66 ± 0.08	0.83715
25.0	0.939 ± 0.022	0.96993	3.09 ± 0.13	0.90374
48.5	1.65 ± 0.028	0.98844	5.90 ± 0.24	0.93413

Table S3: The rates (mM s^{-1}) of reaction for N9- and N3-Bn obtained with increasing concentration of BnCl, using the initial rates method, in the presence of TEA. The mole ratio of BnCl:TEA is 1:1, with [Ade] = 13.5 mM, in DMSO-d_6 at 295 K

	N9-Bn		N3-Bn	
[BnCl] (mM)	$\times 10^{-3}$ mM s^{-1}	R^2	$\times 10^{-3}$ mM s^{-1}	R^2
13.8	0.804 ± 0.016	0.9714	0.237 ± 0.008	0.9172
27.6	2.30 ± 0.09	0.9540	0.659 ± 0.043	0.8681
55.2	3.52 ± 0.06	0.9932	1.53 ± 0.14	0.8863
82.8	5.32 ± 0.13	0.9927	1.62 ± 0.03	0.9853
110	6.22 ± 0.11	0.9940	2.38 ± 0.09	0.9576

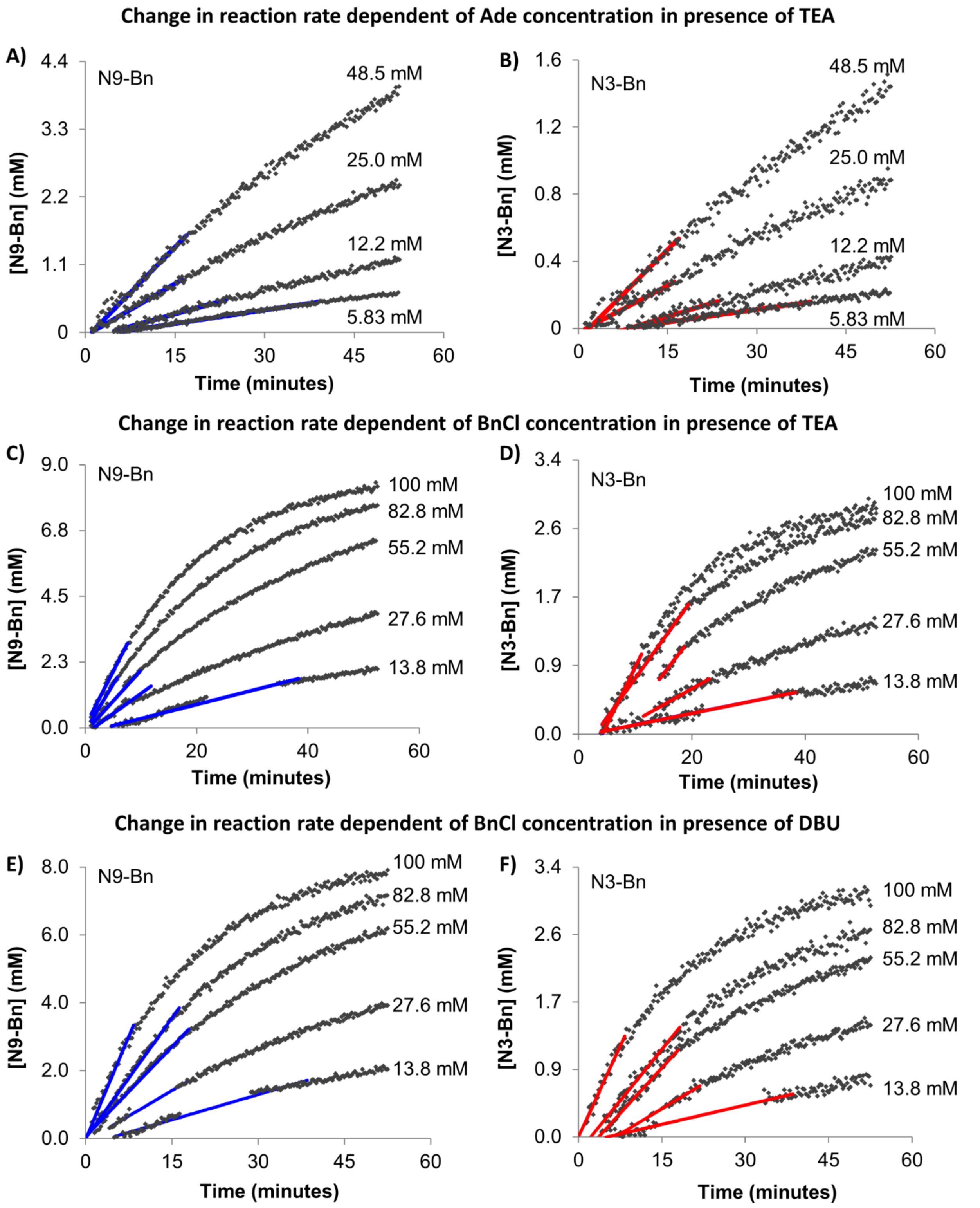

Figure S4: Concentration vs time plots of N9-Bn (blue circles) and N3-Bn (red circles) with the change in [Ade] (A and B), [BnCl:TEA] in a 1:1 ratio (C and D), and [BnCl:DBU] in a 1:1 ratio (E and F) concentration in DMSO-d_6 at 295 K. Varying concentration is indicated in mM in the figure.

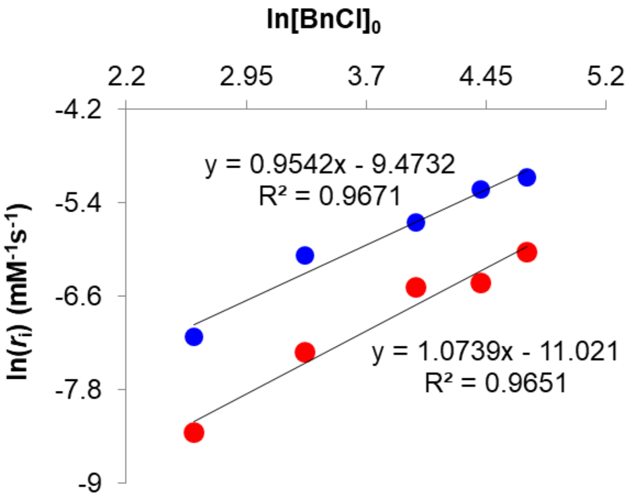

Figure S5: The ln(r_i) against ln$[BnCl]_0$ at concentrations of BnCl:TEA (1:1) of 13.8, 27.6, 55.2, 82.8, and 110 mM, in DMSO-d_6 at 295 K with Ade at 13.5 mM, for N9-Bn (blue circles) and N3-Bn (red circles).

Table S4: The rates (mM s^{-1}) of reaction for N9- and N3-Bn obtained with increasing concentration of BnCl, using the initial rates method, in the presence of DBU. The mole ratio of BnCl:DBU is 1:1, , with [Ade] = 13.5 mM, in DMSO-d_6 at 295 K

	N9-Bn		N3-Bn	
[BnCl] (mM)	$\times 10^{-3}$ mM s^{-1}	R^2	$\times$ 10^{-3} mM s^{-10}	R^2
13.8	0.826 ± 0.012	0.9838	0.270 ± 0.009	0.9377
27.6	1.66 ± 0.035	0.9902	0.700 ± 0.049	0.9337
55.2	2.94 ± 0.04	0.9895	1.28 ± 0.03	0.9761
82.8	3.95 ± 0.05	0.9927	1.44 ±0.04	0.9676
110	6.70 ± 0.11	0.9925	2.54 ± 0.04	0.9913

Reaction kinetics – Temperature dependent experiments

Figure S6 shows the slope values obtained by plotting the [N9-Bn] against [N3-Bn], which represents k_9/k_3, at various temperatures in the presence and absence of 15C5.

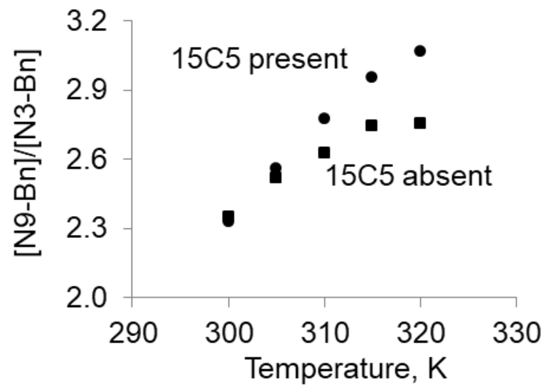

Figure S6: Plot of the ratio of [N9-Bn]/[N3-Bn] vs temperature for the benzylation of adenine using BnCl in the presence (circles) and absence (squares) of 15C5 in DMSO.

Figure S7 and S8 show the [BnCl] vs time plots and the residual plots from the linear fit of the initial portion of the graphs to eqn 6 (main text) which were used to obtain *k’*, Table 2 in main text.

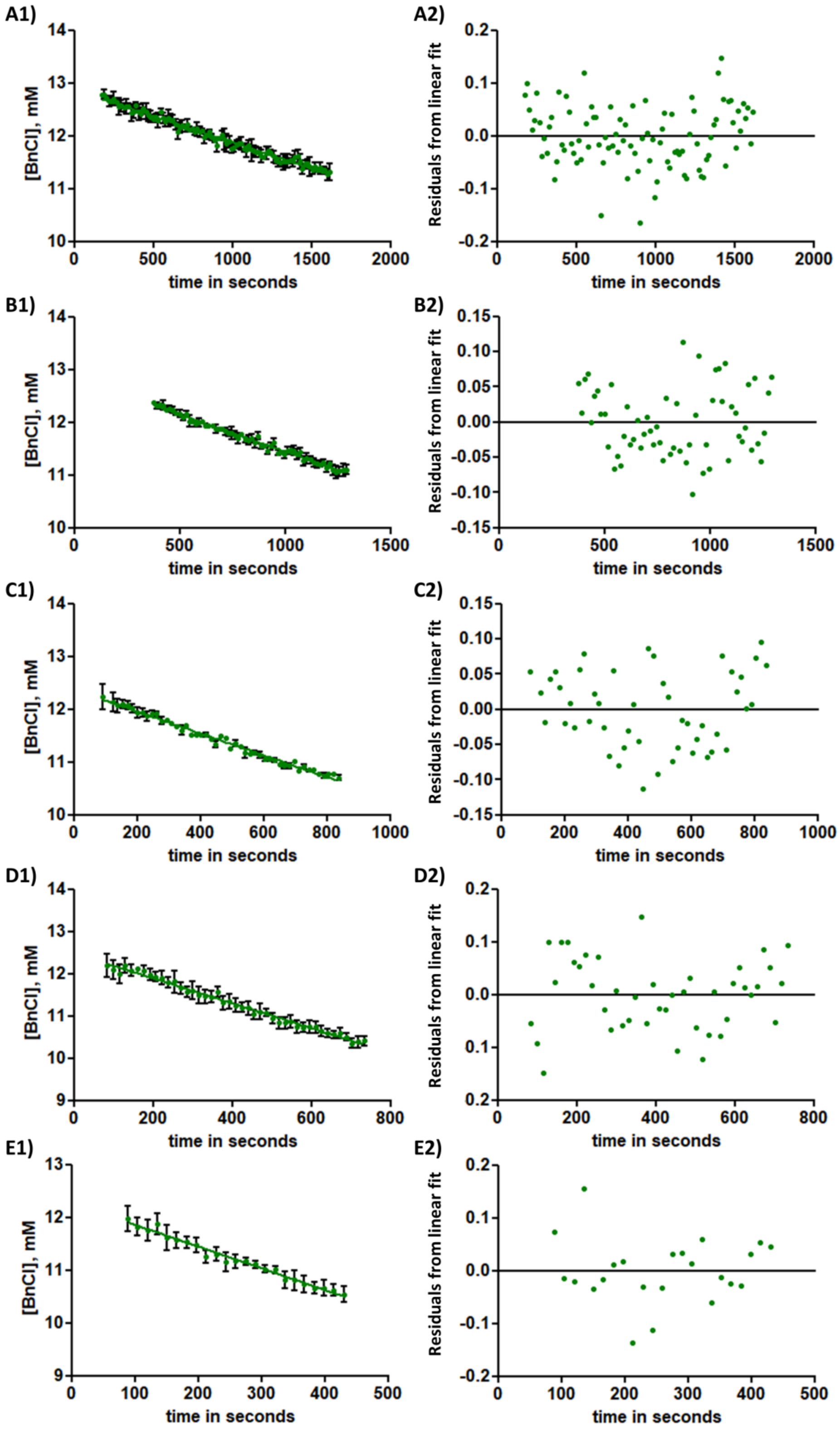

Figure S7: [BnCl] vs time plots and corresponding residual plots for the initial rates method, at each temperature A) 300, B) 305, C) 310, C) 315 and D) 320 K

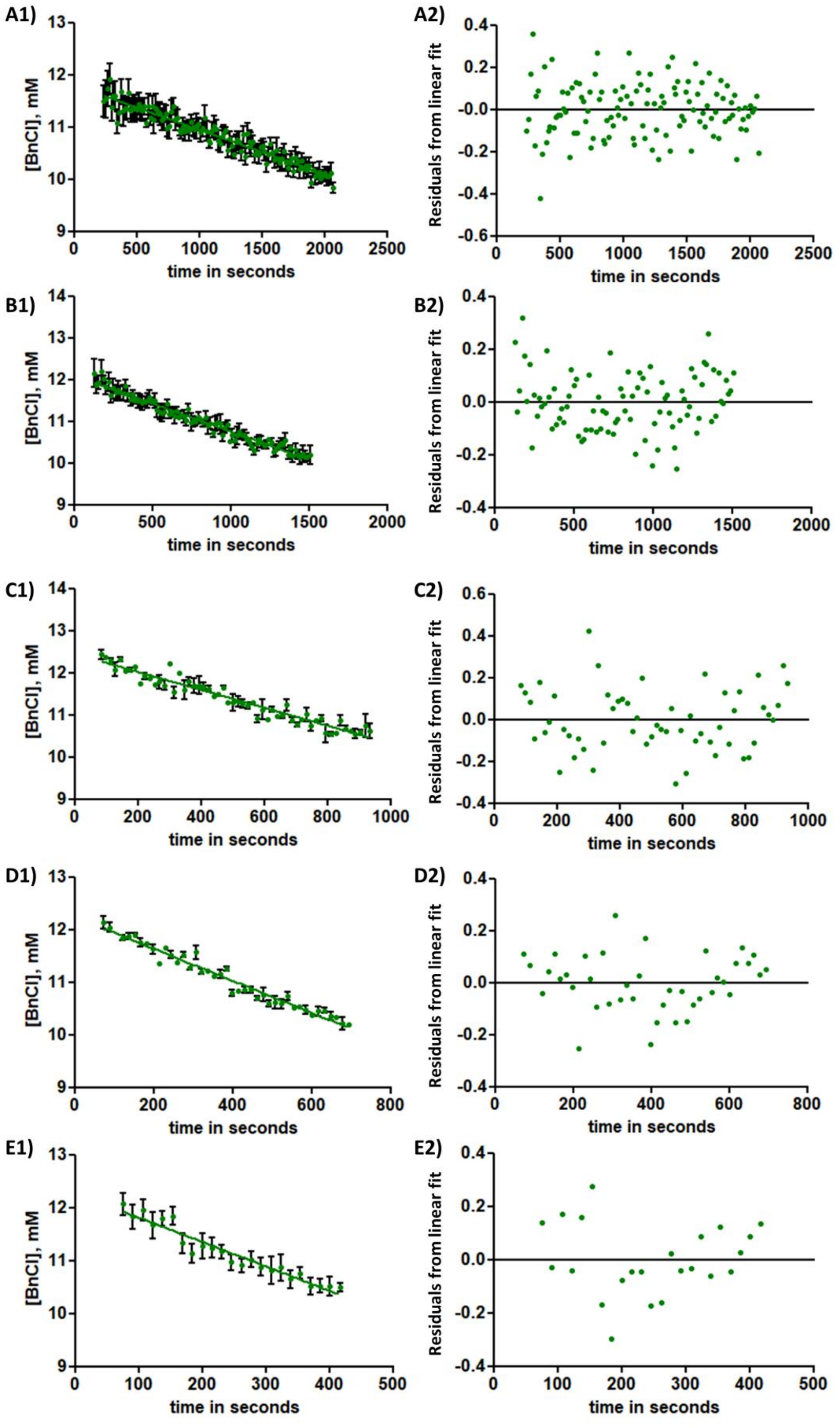

Figure S8: [BnCl] vs time plots and corresponding residual plots for the initial rates method at each temperature, at A) 300, B) 305, C) 310, C) 315 and D) 320 K, with 15C5

Figure S9 and S10 show the [N9-Bn] and [N3-Bn] vs time plots and the residual plots from the linear fit of the initial portion of the graphs to eqn 1 which were used to obtain k_9 and k_3, Table S5.

$$\frac{\Delta[\text{N-Bn}]}{\Delta t} = (k)[\text{Ade}]_0[\text{BnCl}]_0, \quad (1)$$

where N-Bn is N9- or N3-Bn and k is k_9 or k_3.

Table S5: The experimentally determined rate constants ($M^{-1}s^{-1}$) for the formation of N9-Bn (k_9) and N3-Bn (k_3), in the absence and presence of 15-crown-5 ether.

	N9-Bn	N3-Bn
Temperature (K)	$10^{-2}\,k_9$	$10^{-2}\,k_3$
300	0.699 ± 0.015	0.284 ± 0.017
305	1.15 ± 0.03	0.434 ± 0.027
310	1.58 ± 0.04	0.542 ±0.019
315	2.21 ± 0.08	0.748 ± 0.029
320	3.08 ± 0.07	0.993 ± 0.038
	15-crown-5 ether present	
300	0.680 ± 0.012	0.275 ± 0.015
305	1.02 ± 0.02	0.443 ± 0.045
310	1.87 ± 0.07	0.708 ± 0.052
315	2.31 ± 0.07	0.841 ± 0.041
320	3.34 ± 0.23	1.18 ± 0.09

[Ade] = 6.65 mM, [BnCl] = 14.1 mM

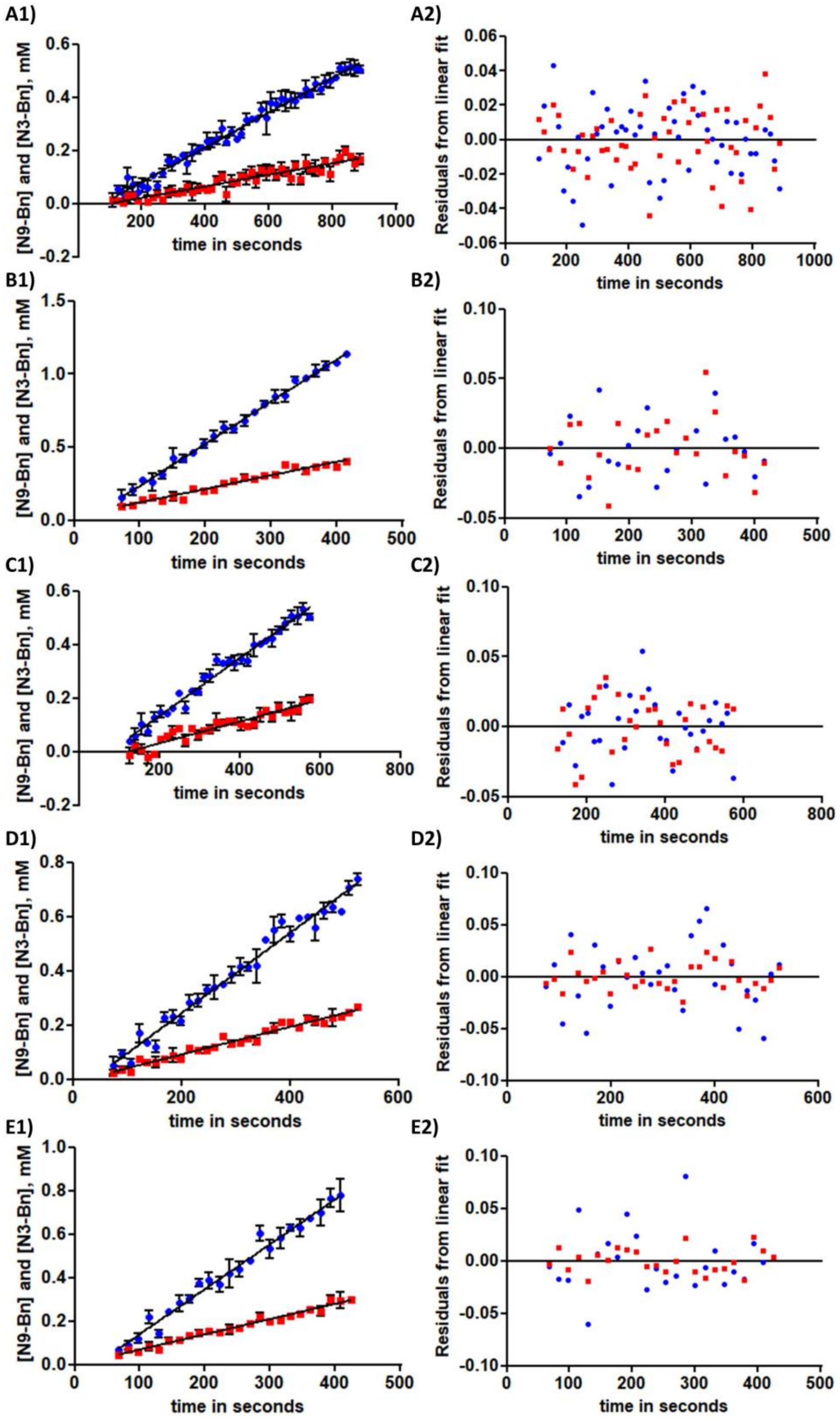

Figure S9: [N9-Bn] (blue circles) and [N3-Bn] (red circles) vs time plots and corresponding residual plots for the initial rates method at each temperature, A) 300, B) 305, C) 310, C) 315 and D) 320 K

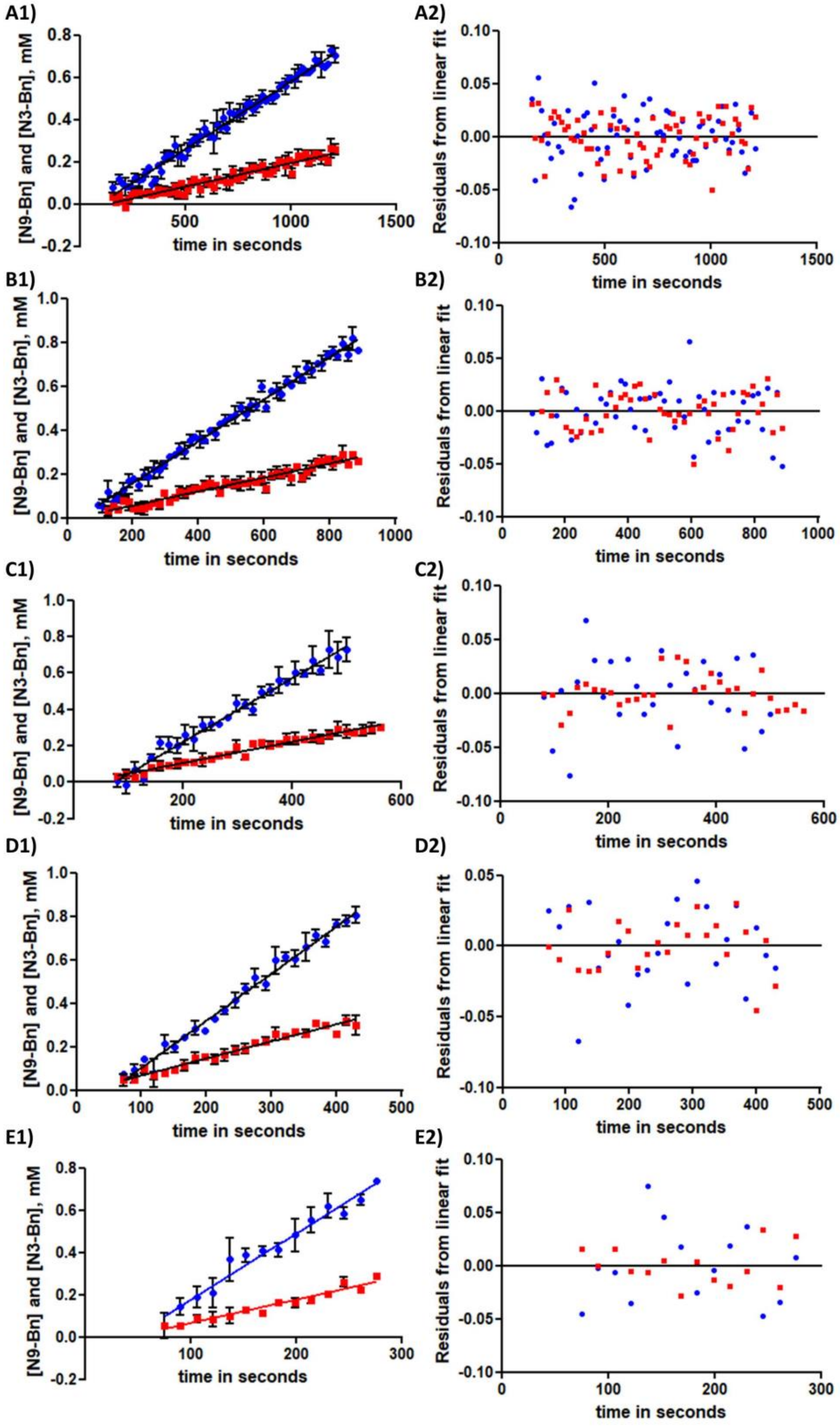

Figure S10: [N9-Bn] (blue circles) and [N3-Bn] (red circles) vs time plots and corresponding residual plots for the initial rates method at each temperature, A) 300, B) 305, C) 310, C) 315 and D) 320 K with 15C5

Arrhenius and activation parameters for N9-Bn and N3-Bn

The plots of ln *k* against 1/T for the formation of N9- and N3-Bn in the absence and presence of 15C5 ether are shown in Figure S11 A and B, respectively. Figure S11 C and D show the Eyring plots for the formation of N9-Bn and N3-Bn in the absence and presence of 15C5, respectively, in anhydrous DMSO-d_6 for the temperature range.

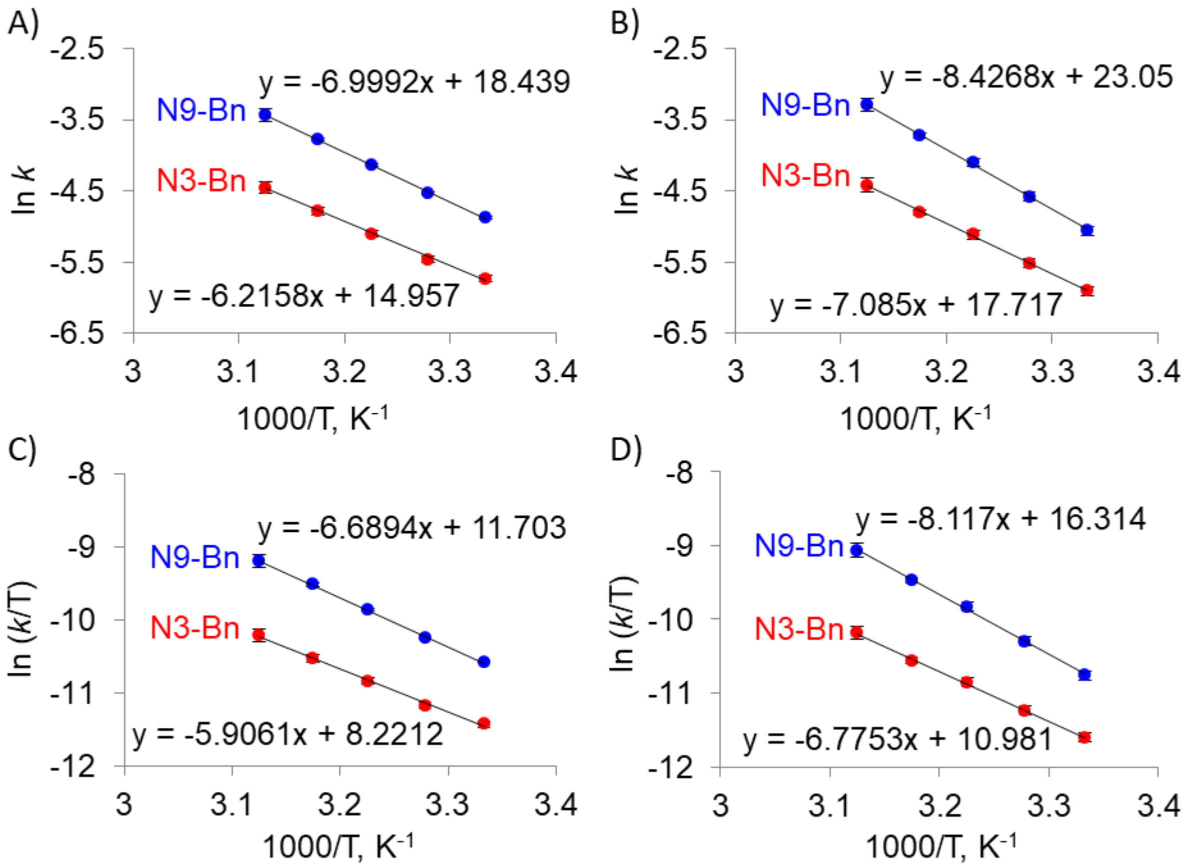

Figure S11: The Arrhenius plot of ln *k* against 1/T for the formation of N9-Bn (blue circles) and N3-Bn (red circles) in the absence (A) and presence of 15C5 (B). The Eyring plots for the formation of N9-Bn (blue circles) and N3-Bn (red circles) in the C) absence and D) presence of 15C5. Reactions are performed in anhydrous DMSO-d_6 for the temperature range 300 – 320 K, increasing in 5 K intervals.

Computational analysis of the reaction pathway

The change in the ΔE_{ZPVE} for the N1, N3, N7 and N9 reaction profiles in the absence and presence of the Na^+ ion are shown in Figure S12.

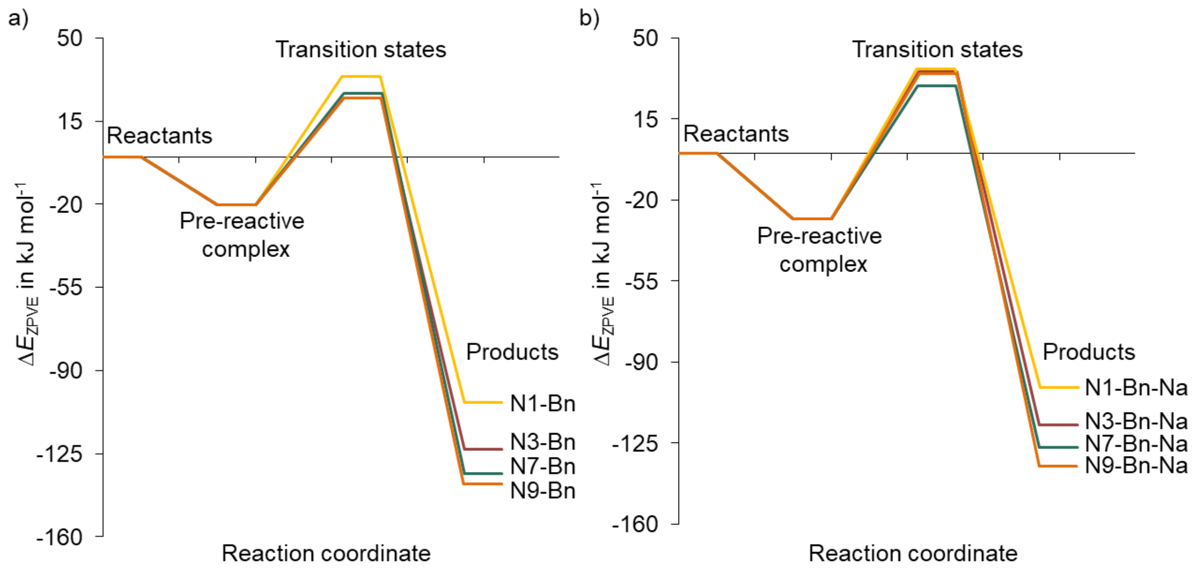

Figure S12: The ΔE_{ZPVE} along the reaction path for the formation of N1-, N3-, N7- and N9-Bn in a) the absence and b) in the presence of the Na^+ ion.

The activation energies for all the possible reaction paths were calculated as the difference between the Gibbs free energy of the transition state complexes and the sum of the Gibbs free energy of the reactants. The rate constant was calculated as follows:

$$k = \frac{k_B T}{h c_0} e^{\left(\Delta G^{\ddagger}/RT\right)} \qquad (2)$$

Effect of explicit solvent molecules:

To study the effect of explicit solvent molecules on the dissociation of the ion pair, two scans have been performed in which the Na^+ is moved away from the adeninate anion. The first scan involves the complex with the sodium ion chelated at the N3N9 position (Na-Ade(N3N9)) in which the implicit solvent system is used to incorporate the DMSO solvent. The ΔE along the reaction coordinate d(N9,Na^+) is shown in Figure S13. The second scan incorporates 4 explicit DMSO solvent molecules, Na-Ade(N3N9)-4DMSO, Figure S14.

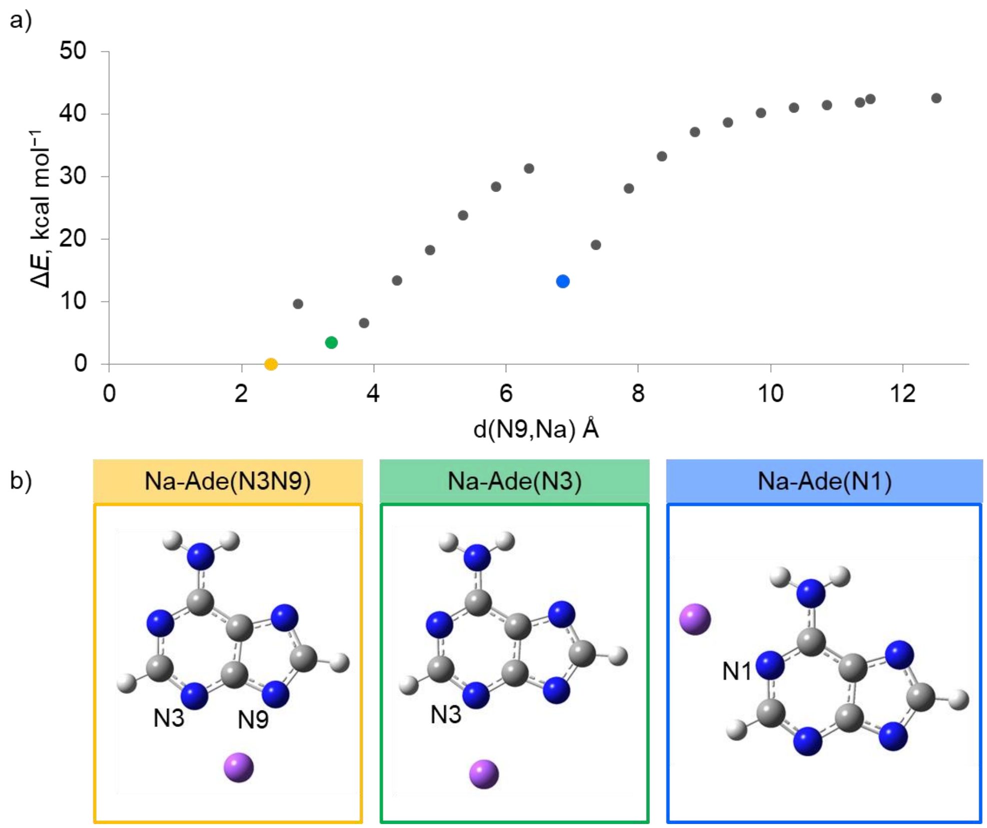

Figure S13: a) ΔE (kJ mol^{-1}) along the reaction coordinate d(N9, Na) in Å, resulting from scanning the Na^+ ion away from the adeninate anion of the Na-Ade(N3N9) complex, and b) optimized structures for the yellow, green and blue points shown in (a).

In Figure S13, to fully remove the Na^+ counter ion, the molecular system experiences an energy penalty of 43 kJ mol^{-1}. As the Na^+ ion is moved further away from the N3N9 site of the adeninate anion it is attracted to the N3 and then the N1 nitrogen atom. From the N3N9 position (yellow point) the system is stabilized along the reaction coordinate which corresponds to complexation at the N3 and N1 atom (green and blue point, respectively).

To fully remove the Na^+ counter ion from the adeninate anion in the presence of the four DMSO solvent molecules, the molecular system pays a penalty of 15 kJ mol^{-1}, Figure S14. Thus, the presence of explicit solvent molecules lowers the energy required to dissociate the sodium chelated adeninate complex by −28 kJ mol^{-1} relative to the system studied using implicit DMSO solvent. This is due to the favourable interactions between the DMSO molecules and the Na^+ counter ion. In Figure S14, the three highly stabilized energy structures are shown. The molecular system is largely stabilized relative to the starting structure (yellow dot) by −40 kJ mol^{-1} as the counter ion complexes simultaneously at the N1 atom and with three DMSO solvent molecules (green point). As the Na^+ is scanned away from the N1 atom, three DMSO solvent molecules migrate with the ion, stabilizing the system again by −10 kcal mol (blue point) relative to the starting structure.

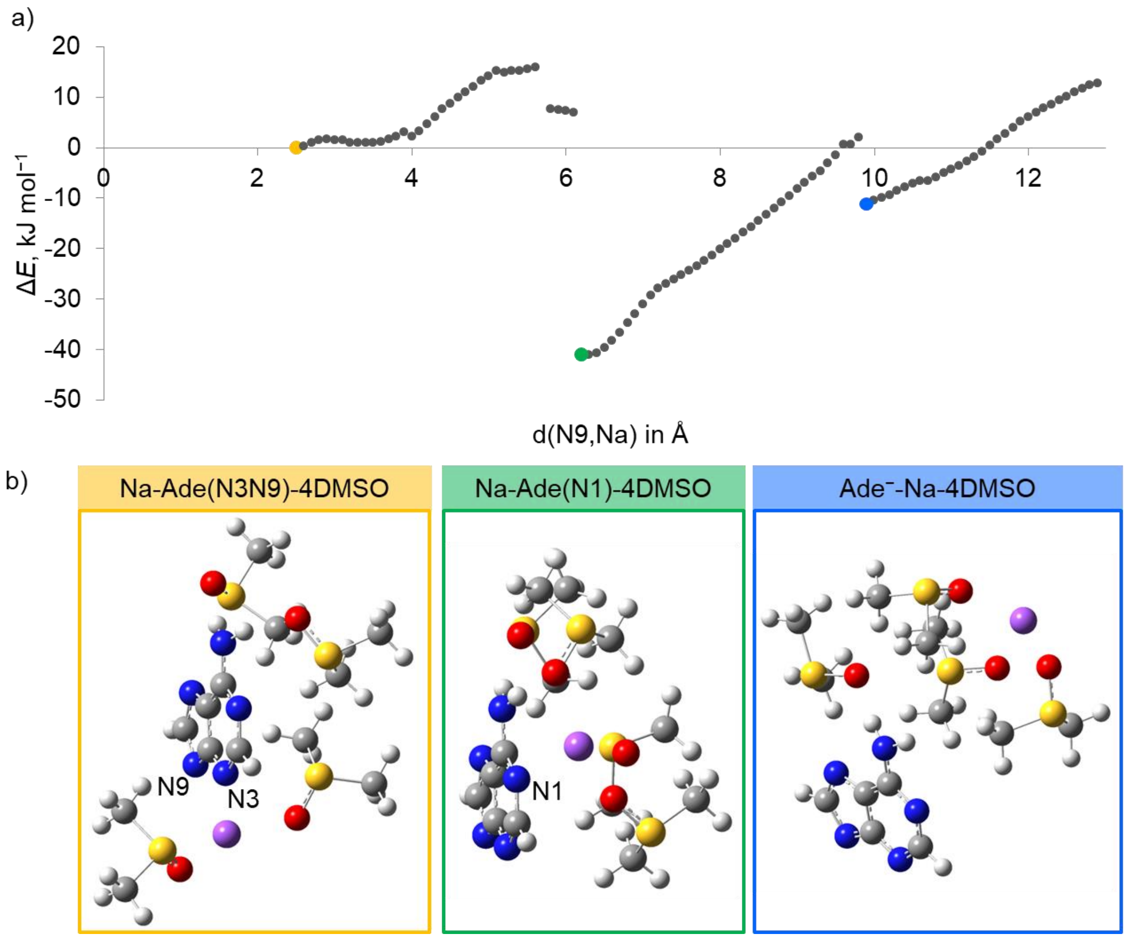

Figure S14: a) ΔE (kJ mol^{-1}) along the reaction coordinate d(N9, Na) in Å, resulting from scanning the Na^+ ion away from the adeninate anion of the Na-Ade(N3N9)-4DMSO complex, and b) optimized structures for the yellow, green and blue points shown in (a).

Hence, if the benzylation of the adeninate anion is to be studied in the presence of the Na^+ counter ion, the DMSO molecules will need to be included as they will greatly reduce the amount of energy required to shift the Na^+ ion out of place as the electrophile is approached by N3 and N9 atoms of the purine ring. This could explain why, in this and previous research, the N9:N3:N7 product ratio is unaffected by the change in counter ion (K^+, Na^+ and Li^+) in polar aprotic solvents.[1] The polar protic solvents can potentially play a critical role in facilitating the removal of the counter ion,

reducing its influence on the alkylation pattern. Furthermore, the DMSO solvent molecules form highly favourable hydrogen bonds with the NH_2 group of the adeninate anion, increasing the energy barrier for benzylation at the N1 and N7 atoms.

The entropy-enthalpy compensation effect

It is well-known in literature that the rate constants of two comparable reactions may be similar although one of them has both, a higher activation enthalpy and a higher (usually less negative) activation entropy.[2] This leads to a crossing of the two rate constants at an isokinetic temperature $T_i = \Delta H^{\ddagger}/\Delta S^{\ddagger}$ $(\Delta G^{\ddagger} = 0)$ within or outside the experimental window in an Arrhenius representation (Figure S15a). This is known as the compensation effect and is attributed to an association pre-equilibrium (formation of a pre-equilibrium complex) which involves an energetic minimum ΔH_A (index A for association or adsorption) in front of the activation barrier (Figure S15b).

In the present case it is proposed that the pre-equilibrium corresponds to a stabilizing association of the reaction complex R_1, consisting of the adeninate anion, the sodium cation, and the benzyl chloride, with the crown ether R_2, under formation of a complex associated state ($R_1 \cdots R_2$, Figure S16) followed by the desired reaction to the product:

$$R_1 + R_2 \leftrightarrow R_1 \cdots R_2 \rightarrow \text{product} \quad (3)$$

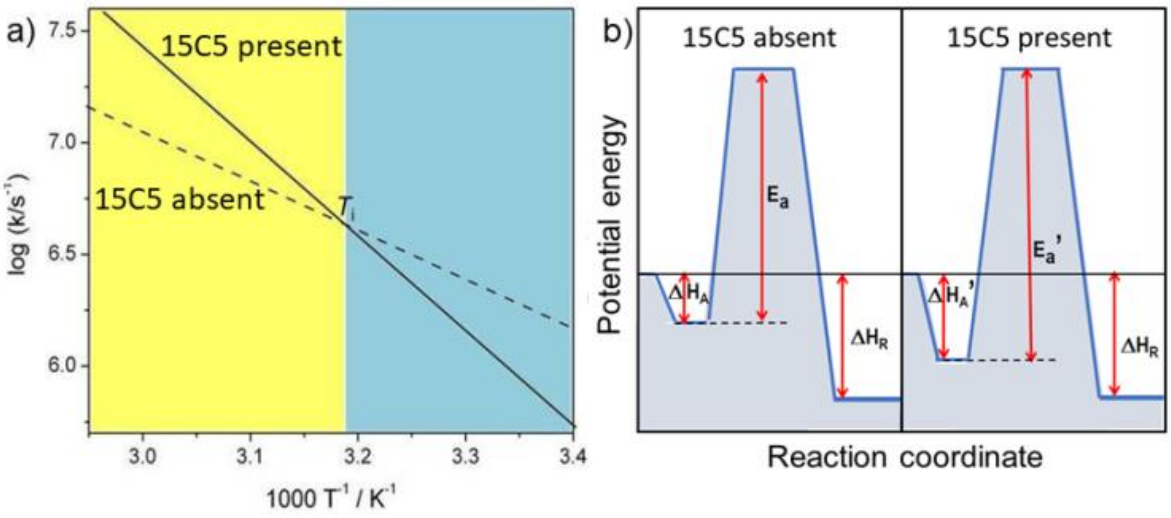

Figure S15. Schematic visualisation of the effect of an association pre-equilibrium with association enthalpy ΔH_A. a) shows the Arrhenius plot and b) the reaction coordinate diagram for the elementary catalytic reaction step with activation energy E_a in the absence of association with 15C5 (dashed line, left part of b)) and the compensation effect in the presence of 15C5 with enhanced Arrhenius pre-exponential factor and activation energy $E_a' = E_a+|\Delta H_A|$ (solid line, right side of b)). In the Arrhenius plot, the two curves cross at the isokinetic temperature T_i. For $T<T_i$ (blue shaded regime) the pre-equilibrium reduces the rate constant because there is significant association, for $T>T_i$ (yellow shaded regime) the reaction is dominated by the free reactants (Figure adapted from Ref. 2).

The rate-determining step that starts from this minimum requires a higher activation energy E_a' but provides already part of the association and thus requires a less negative activation entropy.

$$K_A = \frac{[R_1 \cdots R_2]}{[R_1][R_2]} = \exp\left(-\frac{\Delta G_A}{RT}\right) = \exp\left(\frac{\Delta S_A}{R}\right)\exp\left(-\frac{\Delta H_A}{RT}\right) . \quad (4)$$

The pre-equilibrium changes the initial rate constant of eqn. (8) (main text) to

$$k_{obs} = \frac{k_B T}{hc_0} K_A \exp\left(-\frac{\Delta G^{\ddagger}}{RT}\right) = \frac{k_B T}{hc_0} \exp\left(-\frac{\Delta H^{\ddagger}+\Delta H_{\mathrm{A}}}{RT}\right) \exp\left(\frac{\Delta S^{\ddagger}+\Delta S_{\mathrm{A}}}{R}\right) \quad (5)$$

in which the experimental activation energy $E_a = \Delta H^{\ddagger} + RT + |\Delta H_A|$ includes not only the activation enthalpy in the absence of the crown ether but also the enthalpy of the association pre-equilibrium. In the same sense, the pre-exponential factor, $\ln(ek_B T/h) + \Delta S_A/R + \Delta S^{\ddagger}/R$, contains the association entropy with the sodium ion. Since the adsorption enthalpy and entropy are both negative, the adsorption pre-equilibrium increases both, the apparent Arrhenius activation energy and the corresponding pre-exponential factor.

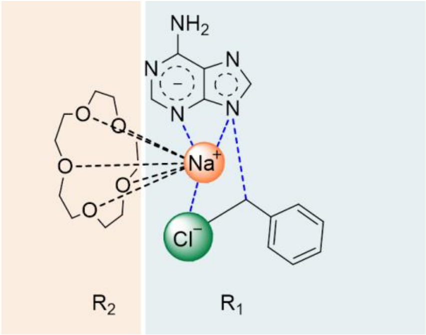

Figure S16. The pre-equilibrium complex consisting of the adeninate anion, the sodium cation, benzyl chloride, and the crown ether, 15C5.

The behaviour observed in Table 4 (main text) by comparison of the parameters in the absence and the presence of the 15C5 crown ether reveals an increase in the $\Delta H^{\ddagger}$ of both the N9- and N3-Bn in the presence of 15C5 that is compensated for by the

increase in $T\Delta S^{\ddagger}$. As a result, there is no change in the $\Delta G^{\ddagger}_{300}$ term. The inversion temperature is close to 300 K, and the measurements represent the yellow-shaded part of Figure 6a. A plot of the $\Delta H^{\ddagger}$ vs $T\Delta S^{\ddagger}$ for both the N9- and N3-Bn, Figure S17, shows slopes close to unity, indicating almost full compensation between $\Delta H^{\ddagger}$ and $T\Delta S^{\ddagger}$. Although there are only two points for each plot, the entropy-enthalpy compensation effect is significant. This in turn can explain the indifferent product ratios in previous work when various alkyl halides were used[1,3] and when different counter ions were present,[1] however, this will need to be confirmed with further experimental work.[4]

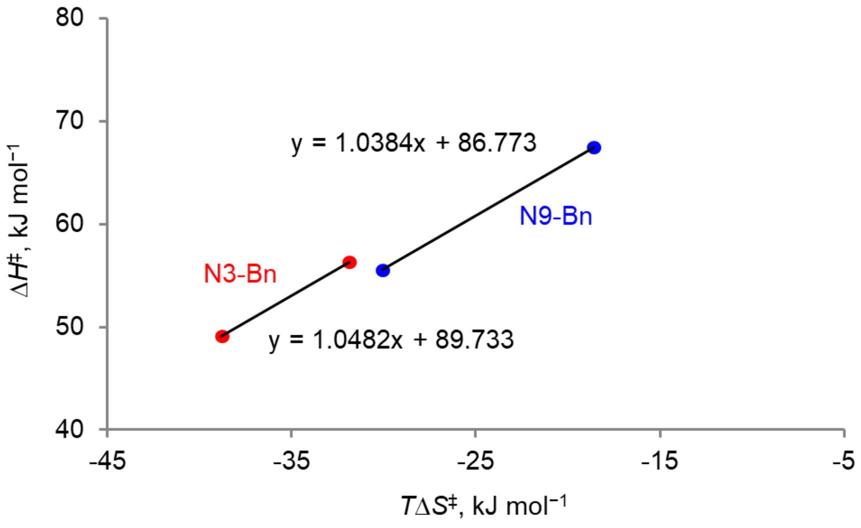

Figure S17. $\Delta H^{\ddagger}$ vs. $T\Delta S^{\ddagger}$ enthalpy-entropy compensation plot for the formation of N9-Bn (blue circles) and N3-Bn (red circles) in the absence and presence of 15C5 in anhydrous DMSO at 300 K.

The difference in the thermodynamic parameters of the reaction in the absence and the presence of the crown ether reflects the enthalpy and entropy of the association

reaction of 15C5 with Na^+. It is plausible that this complexation reduces the extent of blocking at the adeninate nitrogen reaction site and gives better access to the formation of a pre-complex with the benzyl chloride.

References

[1] M. Rasmussen, J. Hope, *Aust. J. Chem.* **1982**, *35*, 535.

[2] E. Roduner, *Chem. Soc. Rev.* **2014**, *43*, 8226–8239.

[3] M. Rasmussen, J. Hope, *Aust. J. Chem.* **1982**, *35*, 525.

[4] A. B. Koudriavtsev, W. Linert, *Match-Commun. Math. Comput. Chem.* **2013**, *70*, 7–28.
